# Pleiotropic, Unique and Shared Responses Elicited by IL-6 Family Cytokines in Human Vascular Endothelial Cells

**DOI:** 10.3390/ijms23031448

**Published:** 2022-01-27

**Authors:** Madelene Lindkvist, Mulugeta M. Zegeye, Magnus Grenegård, Liza U. Ljungberg

**Affiliations:** Cardiovascular Research Centre (CVRC), School of Medical Sciences, Örebro University, 70362 Örebro, Sweden; madelene.lindkvist@oru.se (M.L.); mulugeta.m.zegeye@oru.se (M.M.Z.); magnus.grenegard@oru.se (M.G.)

**Keywords:** cardiotrophin-1 (CT-1), ciliary neurotrophic factor (CNTF), CCL23, chemotaxis, interleukin-11 (IL-11), leukemia inhibitory factor (LIF), hepatocyte growth factor (HGF)

## Abstract

Vascular endothelial cells express glycoprotein 130 (gp130), which is utilized as a signaling receptor by cytokines in the interleukin-6 (IL-6) family. Several IL-6 family cytokines can be found in the circulatory system during physiological or pathological conditions, and may influence endothelial function and response. This study evaluated and compared the cellular and molecular responses induced by IL-6 family cytokines in human endothelial cells. A proteomic analysis showed that IL-6 family cytokines induce the release of a range of proteins from endothelial cells, such as C-C motif chemokine ligand 23, hepatocyte growth factor, and IL-6. Pathway analysis indicated that gp130-signaling in endothelial cells regulates several functions related to angiogenesis and immune cell recruitment. The present investigation also disclosed differences and similarities between different IL-6 family cytokines in their ability to induce protein release and regulate gene expression and intracellular signaling, in regards to which oncostatin M showed the most pronounced effect. Further, this study showed that soluble gp130 preferentially blocks trans-signaling-induced responses, but does not affect responses induced by classic signaling. In conclusion, IL-6 family cytokines induce both specific and overlapping molecular responses in endothelial cells, and regulate genes and proteins involved in angiogenesis and immune cell recruitment.

## 1. Introduction

The vascular endothelium plays a crucial role in maintaining vascular homeostasis. Due to their strategic location on the luminal side of all blood vessels, vascular endothelial cells are in constant contact with circulating blood, and are continuously exposed to numerous circulating mediators, such as cytokines and chemokines. Cytokines are important signaling molecules, released into the blood and other tissues, that mediate their effect by binding to specific surface receptors. Glycoprotein 130 (gp130) is a co-receptor expressed on most cell types that acts as a common signaling transducer for cytokines in the IL-6-family, including IL-6, IL-11, oncostatin M (OSM), leukemia inhibitory factor (LIF), cardiotrophin 1 (CT-1), cardiotrophin-like cytokine (CLC), ciliary neurotrophic factor (CNTF), and IL-27 [1] These cytokines affect a variety of cells and have a wide range of cellular functions. In vascular endothelial cells, IL-6 family cytokines have been shown to affect the release of inflammatory mediators, the expression of adhesion molecules, migration, and proliferation [2,3].

**Figure 1:**
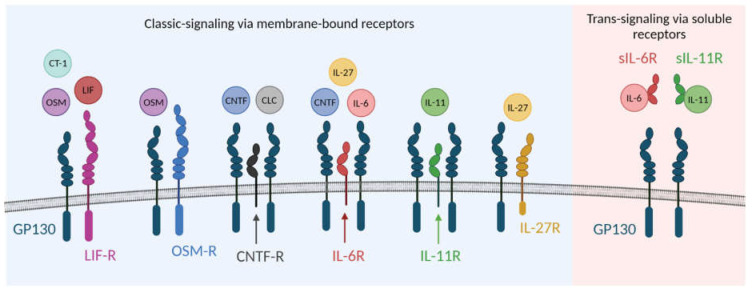
Schematic illustration of receptors used by IL6-family cytokines. Created using BioRender.com accessed on 20 December 2021.

The IL-6 family cytokines bind specific surface receptors (alpha receptors), which, upon binding, dimerize with gp130 to induce an intracellular signal [4] (Figure 1). The IL-6 receptor, IL-11 receptor, and CNTF receptor lack intracellular signaling domains, and are dependent on signaling via gp130.The OSM receptor, LIF receptor, and IL-27 receptor, on the other hand, have signaling capability, but require dimerization with gp130 in order to transduce a signal [1,5]. In addition to the classic cytokine signaling induced via surface receptors, some of the IL-6 family cytokines can also act via soluble receptors. These receptors form a soluble cytokine-receptor-complex that can interact with gp130 on the cell surface. This also facilitates cytokine signaling in cells that lack cytokine-specific receptors but express gp130. This phenomenon is referred to as trans-signaling, and has been shown for IL-6 and IL-11 [5,6]. The soluble receptors can be produced either by alternative splicing or proteolytic cleavage of membrane bound receptors [7,8,9,10].

Signaling via gp130 typically induces activation of the JAK/STAT signaling pathway, but other signaling pathways, such as the PI3-K/AKT or MEK/ERK pathways, are also activated in various cell types [11,12,13]. Cellular specificity of a signaling response for IL-6 family cytokines is primarily dependent on the presence of alpha receptors on the cell surface. In previous studies, we reported that IL-6-induced intracellular signaling and functional outcomes differs significantly when comparing classic signaling to trans-signaling [14,15]. Whether there are qualitative or quantitative differences between the intracellular cascades activated by different IL-6 family cytokines remains to be investigated.

Several studies have shown altered gp130 signaling in inflammatory diseases such as atherosclerosis, rheumatoid arthritis, and cancer [16,17,18,19]. Although most of the effects of IL-6 family cytokines are attributed to their paracrine activities, they can also act in an endocrine manner, as high levels of IL-6 family cytokines can be found in the circulation, for example, during inflammatory conditions [20,21,22]. Vascular endothelial cells can be affected by these cytokines as they express the gp130 signaling receptor, as well as some alpha receptors [15,23,24]. In addition, there is evidence for receptor cross-reactivity, where alpha receptors are activated by related cytokines. For instance, CNTF has been shown to engage IL-6R-gp130 to induce intracellular signaling cascades [23], and, similarly, OSM can bind to and signal via LIF-R-gp130 [24]. In the current study, we aimed to evaluate and compare cellular and molecular responses induced by IL-6 family cytokines in human umbilical vein endothelial cells (HUVECs). Specifically, we characterized the expression of IL-6 family cytokine receptors on HUVECs and investigated the impact of IL-6 family cytokines on the secretion of proteins involved in inflammation, as well as cardiovascular physiology and pathophysiology. Further, we evaluated how IL-6 family cytokines affect intracellular signaling pathways previously shown be induced by gp130 signaling, and investigated whether soluble gp130 is able to interfere with responses induced by IL-6 family cytokines in HUVECs.

## 2. Results

### 2.1. Expression of Receptors for IL-6 Family Cytokines on Human Endothelial Cells

**Figure 2:**
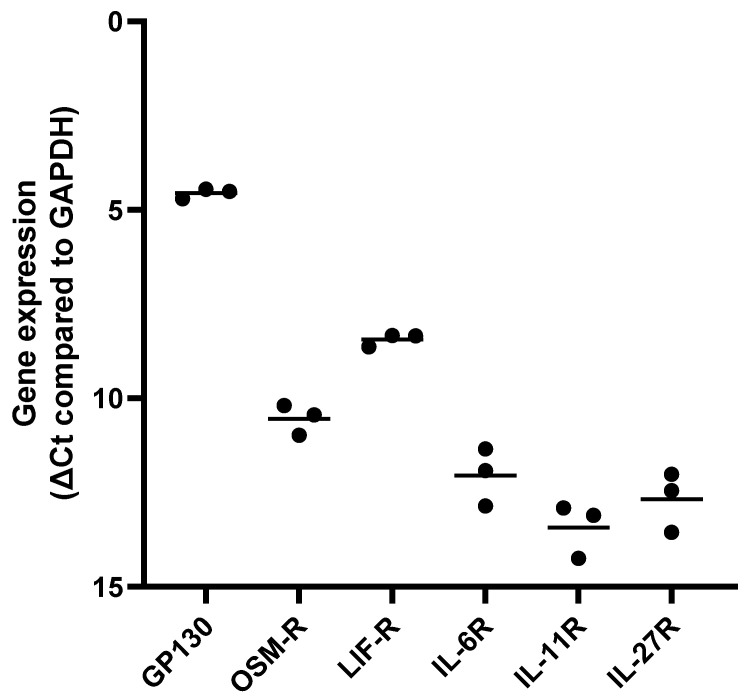
Human umbilical vein endothelial cells express several IL-6 family cytokine receptors. Gene expression of glycoprotein 130 (GP130/IL6ST), oncostatin M receptor (OSM-R), leukemia inhibitory factor receptor (LIF-R), interleukin-6 receptor alpha (IL-6R), interleukin-11 receptor (IL-11R), and interleukin-27 receptor (IL-27R) was analyzed in HUVECs (passage 7–8) cultured under standard conditions. Gene expression was compared to gene expression of GAPDH and presented as ∆Ct compared to GAPDH (**n** = 3).

The expression of receptors utilized by IL-6 family cytokines was evaluated in HUVECs using qPCR. As shown in Figure 2, HUVECs express high levels of gp130 (mean ct 20.5) and LIF-R (mean ct 24.4). They also express medium to low levels of OSM-R (mean ct 26.5), IL-6R (mean ct 28.0), IL-11R (mean ct 29.3), and IL-27R (mean ct 28.6). Gene expression of CNTF-R, on the other hand, was not detected in HUVECs (ct value > 35). Gene expression of most of these receptors was also shown to be influenced by pro-inflammatory mediators such as TNF-α (Supplementary Figure S1).

### 2.2. IL-6 Family Cytokines Induce Release of a Wide Range of Proteins from Human Endothelial Cells

In order to determine suitable doses of cytokines to use, dose-response experiments were performed (Supplementary Figure S2). For subsequent experiments, a fixed dose of 10 ng/mL was used for all cytokines and soluble receptors. To characterize the impact of IL-6 family cytokines on the release of proteins from HUVECs, a proteomic analysis was performed. In total, 70 proteins could be detected in the cell culture medium. Protein release induced by OSM, LIF, and CT-1, as well as IL-6 and IL-11 (classic and trans-signaling), which showed a very similar pattern (Table 1). CNTF had a limited effect on protein release (Table 1), while no effects were seen with CLC and IL-27 (Supplementary Materials, Table S1). A full list of all detected proteins is shown in Supplementary Materials. Among the most strongly induced proteins were C-C motif chemokine ligand 23 (CCL23), hepatocyte growth factor (HGF), and IL-6. In addition, OSM was the only cytokine in the IL-6 family that resulted in a downregulation of several proteins compared to untreated controls. Neither sIL-6R nor sIL-11R caused protein release on their own (data not shown).

### 2.3. IL-6 Family Cytokines Regulate Proteins Involved in a Wide Range of Biological Functions

**Figure 3:**
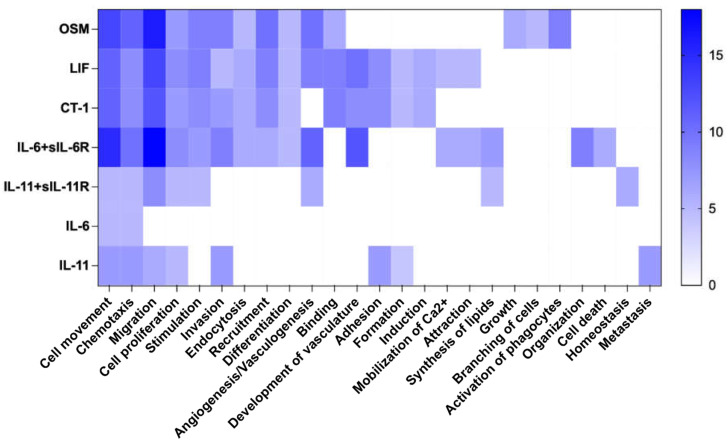
Biological functions regulated by IL-6 family cytokines in human umbilical vein endothelial cells. Using Ingenuity Pathway Analysis, biofunctions regulated by IL-6 family cytokines were evaluated. For these analyses, differentially regulated proteins with a fold change of 1.5 and Benjamini–Hochberg’s estimated false discovery rate <0.2 were included, and functions with z-score of >2 were considered as enriched. The intensity of the color represents number of differentially regulated proteins in each function.

To further elucidate biological functions that are regulated by IL-6 family cytokines in vascular endothelial cells, we performed gene ontology [25] enrichment analysis using Ingenuity Pathway Analysis. A wide range of biological functions were enriched, and there was a large overlap between the different cytokines (Figure 3). In total, 25 different biological functions were found to be enriched, among which cell movement, chemotaxis, migration, and cell proliferation were the top regulated functions. All enriched functions were predicted to be activated by the treatments. A full list of all enriched functions and the regulated proteins in those functions are shown in Supplementary Materials, Table S2.

### 2.4. Transcriptional Regulation of Selected Genes by IL-6 Family Cytokines in Human Endothelial Cells

**Figure 4:**
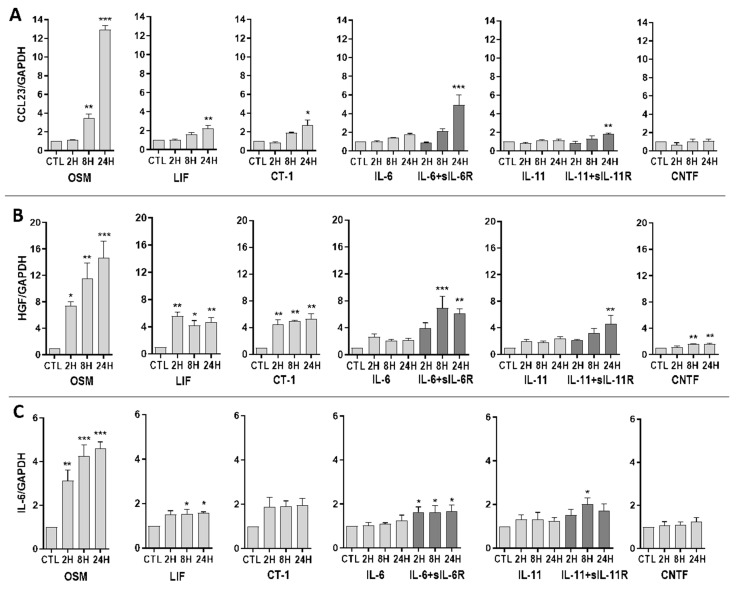
Relative gene expression of CCL23, HGF and IL-6 in human umbilical vein endothelial cells (HUVECs) induced by IL-6 family cytokines. HUVECs were exposed to 10 ng/mL of oncostatin M (OSM), leukemia inhibitory factor (LIF), cardiotrophin 1 (CT-1), interleukin-6 (IL-6), alone or together with soluble IL-6 receptor, interleukin 11, alone or together with soluble IL-11 receptor, or ciliary neurotrophic factor (CNTF) for 2–24 h. Gene expression of (**A**) C-C motif chemokine ligand 23 (CCL23), (**B**) hepatocyte growth factor (HGF), and (**C**) IL-6 was analyzed. Gene expression levels were normalized to gene expression of GAPDH, and untreated control was set to 1. Data are shown as mean and SEM of three independent experiment run in duplicate (* *p* < 0.05, ** *p* < 0.01, *** *p* < 0.001).

Three of the top upregulated proteins identified by proteomic analysis (CCL23, HGF, and IL-6) were further studied. Gene expression of these genes was analyzed after 2–24 h of exposure to IL-6 family cytokines using qPCR. Overall, the results showed a similar pattern compared to the proteomic analysis. Upregulated gene expression of CCL23, HGF, and IL-6 was induced by OSM, LIF, and CT-1, as well as IL-6 and IL-11 in combination with their respective soluble receptors (Figure 4). These effects were the most pronounced after 24 h of treatment. CNTF showed a minor induction of gene expression of HGF, and no effect on gene expression of CCL23 or IL-6. CLC and IL-27 had no effect on gene expression of HGF, CCL23, and IL-6 (data not shown).

### 2.5. IL-6 Family Cytokines Induce Phosphorylation of STAT3, AKT and ERK1/2

**Figure 5:**
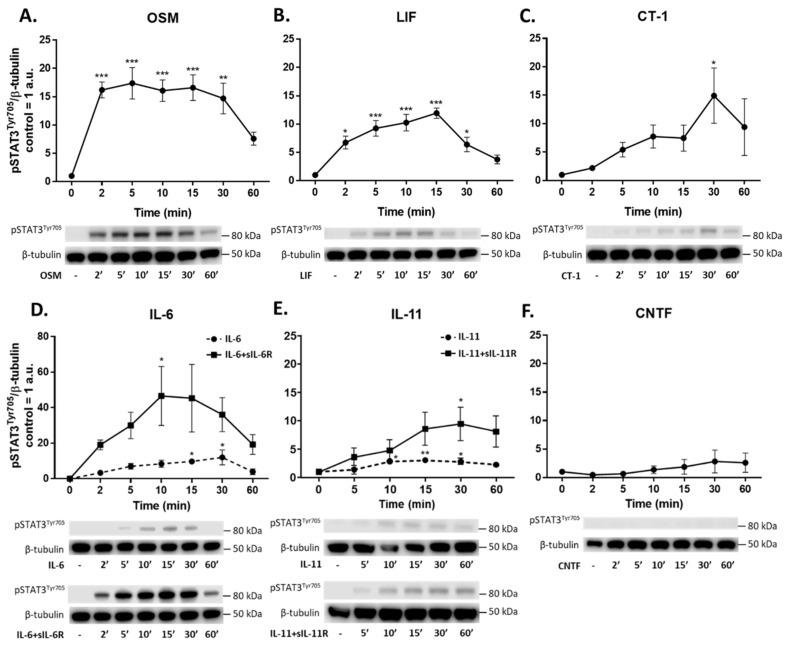
Phosphorylation of STAT3 induced by IL-6 family cytokines in human umbilical vein endothelial cells (HUVECs). Western blot analyses of phosphorylated STAT3 (pSTAT3^Tyr705^) in HUVECs induced by IL-6 family cytokines (10 ng/mL) for 2–60 min. Representative blots and quantifications of **n** = 3 are shown. Control was set to 1; data are normalized to β-tubulin and shown as mean arbitrary units (a.u.) and SEM. * *p* < 0.05, ** *p* < 0.01, *** *p* < 0.001 compared to control. Treatments were: (**A**) oncostatin M (OSM), (**B**) leukemia inhibitory factor (LIF), (**C**) cardiotrophin 1 (CT-1), (**D**) interleukin-6 (IL-6) alone and in combination with soluble IL-6 receptor (IL-6+sIL-6R), (**E**) interleukin-11 (IL-11) alone and in combination with soluble IL-11 receptor (IL-11+sIL-11R), and (**F**) ciliary neurotrophic factor (CNTF).

**Figure 6:**
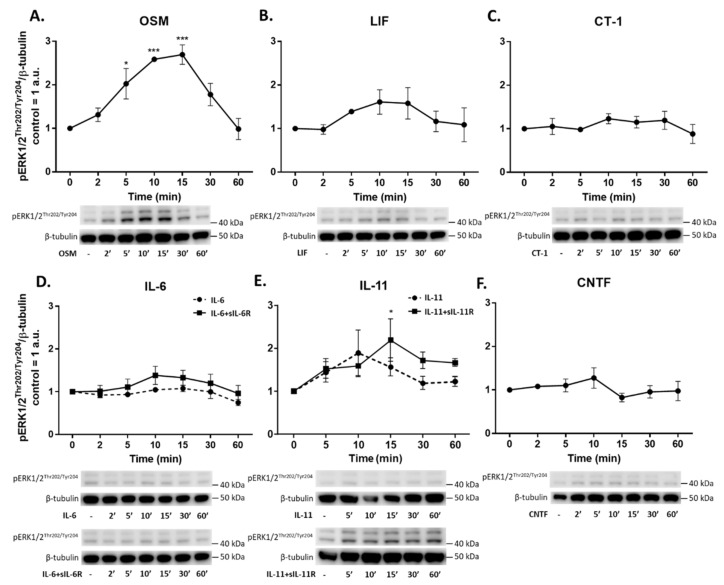
Phosphorylation of ERK1/2 induced by IL-6 family cytokines in human umbilical vein endothelial cells (HUVECs) Western blot analyses of phosphorylated ERK1/2 (pERK1/2^Thr202/Tyr204^) in HUVECs induced by IL-6 family cytokines (10 ng/mL) for 2–60 min. Representative blots and quantifications of **n** = 3 are shown. Control was set to 1; data are normalized to β-tubulin and shown as mean arbitrary units (a.u.) and SEM. * *p* <0.05, *** *p* < 0.001 compared to control. Treatments were: (**A**) oncostatin M (OSM), (**B**) leukemia inhibitory factor (LIF), (**C**) cardiotrophin 1 (CT-1), (**D**) interleukin-6 (IL-6) alone and in combination with soluble IL-6 receptor (IL-6+sIL-6R), (**E**) interleukin-11 (IL-11) alone and in combination with soluble IL-11 receptor (IL-11+sIL-11R), and (**F**) ciliary neurotrophic factor (CNTF).

**Figure 7:**
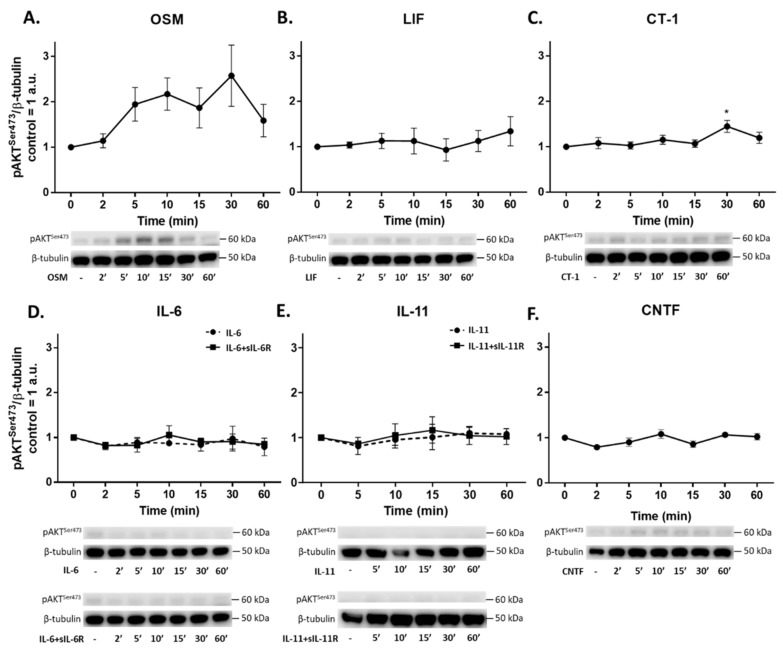
Phosphorylation of AKT induced by IL-6 family cytokines in human umbilical vein endothelial cells (HUVECs). Western blot analyses of phosphorylated AKT (pAKT^Ser473^) in HUVECs induced by IL-6 family cytokines (10 ng/mL) for 2–60 min. Representative blots and quantifications of **n** = 3 are shown. Control was set to 1; data are normalized to β-tubulin and shown as mean arbitrary units (a.u.) and SEM. * *p*< 0.05 compared to control. Treatments were: (**A**) oncostatin M (OSM), (**B**) leukemia inhibitory factor (LIF), (**C**) cardiotrophin 1 (CT-1), (**D**) interleukin-6 (IL-6) alone and in combination with soluble IL-6 receptor (IL-6+sIL-6R), (**E**) interleukin-11 (IL-11) alone and in combination with soluble IL-11 receptor (IL-11+sIL-11R), and (**F**) ciliary neurotrophic factor (CNTF).

To further evaluate the intracellular pathways regulated by IL-6 family cytokines, we performed western blot analyses to detect phosphorylation of STAT3, AKT, and ERK1/2. Phosphorylation of STAT3^Tyr705^ (pSTAT3^Tyr705^) was induced by OSM, LIF, CT-1, IL-11, and IL-6 (Figure 5). Both classic and trans-signaling by IL-11 and IL-6 induced phosphorylation of STAT3; however, trans-signaling (i.e., IL-11/IL-6 combined with their respective soluble receptor) was more effective in this regard. Meanwhile, CNTF did not induce phosphorylation of STAT3. Phosphorylation of ERK1/2^Thr202/Tyr204^ was only seen after exposure to OSM (5–15 min) and IL-11 trans-signaling (at 15 min) (Figure 6). A small but significant phosphorylation of AKT^Ser473^ was induced by exposure to CT-1 for 30 min (Figure 7), while a trend towards AKT phosphorylation could be seen after exposure to OSM. Treatment with CLC and IL-27, as well as sIL-6R and sIL-11R alone, did not result in phosphorylation of either STAT3, AKT, or ERK1/2 (data not shown).

### 2.6. IL-6 Family Cytokines Induce Transcriptional Regulation of SOCS3 in Human Endothelial Cells

**Figure 8:**
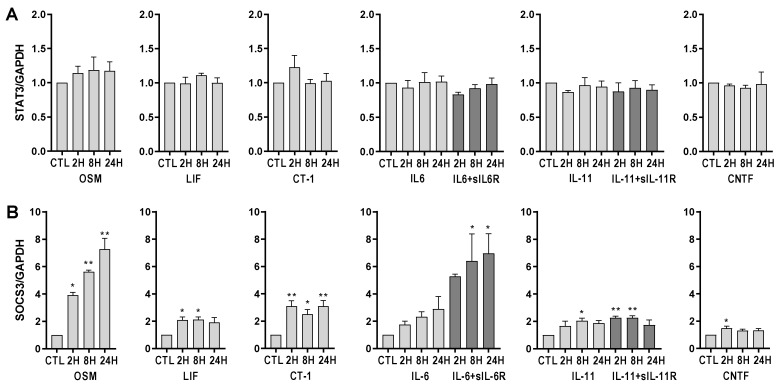
IL-6 family cytokines induces gene expression of SOCS3 but not STAT3 in human umbilical vein endothelial cells (HUVECs). HUVECS were exposed to 10 ng/mL of oncostatin M (OSM), leukemia inhibitory factor (LIF), cardiotrophin 1 (CT-1), interleukin-6 (IL-6) alone or together with soluble IL-6 receptor, interleukin-11 (IL-11) alone or together with soluble IL-11 receptor, or ciliary neurotrophic factor (CNTF) for 2–24 h. Gene expression of (**A**) Signal Transducer And Activator Of Transcription 3 (STAT3) and (**B**) Suppressor Of Cytokine Signaling 3 (SOCS3) was analyzed. Gene expression was normalized to the expression of GAPDH, and untreated control was set to 1. Data are shown as mean and SEM of three independent experiment run in duplicate (* *p* < 0.05, ** *p* < 0.01).

We further analyzed the gene expression of STAT3 and SOCS3, which are known to be regulated by IL-6 family cytokines. As shown in Figure 8, STAT3 is not transcriptionally regulated by IL-6family cytokines. Gene expression of SOCS3, on the other hand, is significantly induced by OSM, LIF, CT-1, IL-6 trans-signaling, and IL-11 classic and trans-signaling, as well as CNTF.

### 2.7. Soluble gp130 Selectively Interferes with Trans-Signaling Mediated Responses in Human Endothelial Cells

**Figure 9:**
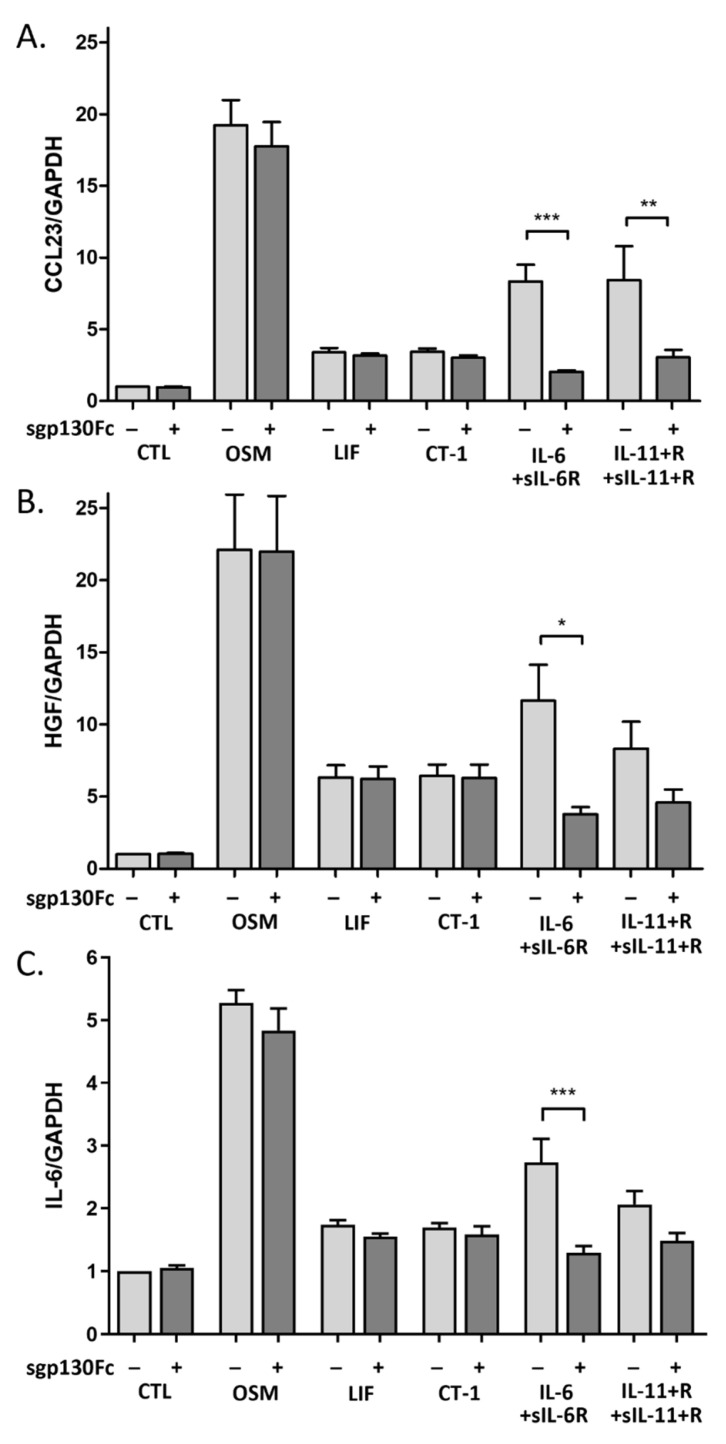
Relative gene expression of human umbilical vein endothelial cells after exposure to IL-6 family cytokines and soluble gp130Fc. Gene expression of (**A**) C-C motif chemokine ligand 23 (CCL23), (**B**) hepatocyte growth factor (HGF), and (**C**) interleukin-6 (IL-6) in response to 10 ng/mL of oncostatin M (OSM), leukemia inhibitory factor (LIF), cardiotrophin 1 (CT-1), IL-6 in combination with soluble IL-6 receptor (IL-6 + sIL-6R), and interleukin-11 (IL-11) in combination with soluble IL-11 receptor (IL-11 + sIL-11R). All treatments were made in the presence or absence of 1 µg/mL soluble gp130Fc (sgp130) for 24h. Gene expression was normalized to the expression of GAPDH, and untreated control (CTL) was set to 1. Data are shown as mean and SEM of three independent experiment run in duplicate (* *p* < 0.05, ** *p* < 0.01, *** *p* < 0.001).

We further investigated whether soluble gp130 (sgp130) interferes with the responses induced by IL-6 family cytokines in HUVECs. OSM, LIF, and CT-1, as well as IL-6 and IL-11 in combination with their respective soluble receptors, were co-incubated with sgp130Fc protein. HUVECs were treated with 10 ng/mL cytokine and 1 µg/mL sgp130Fc for 24 h, and gene expression of CCL23, HGF, and IL-6 were analyzed. IL-6 trans-signaling-induced upregulation of CCL23, HGF, and IL-6 mRNA was almost abolished by sgp130Fc (Figure 9). The upregulation of CCL23 mRNA in response to IL-11 trans-signaling was significantly inhibited by sgp130Fc, while the upregulation of HGF and IL-6 mRNA showed a non-significant reduction. The gene expression of CCL23, HGF, and IL-6 induced by OSM, LIF, and CT-1 was not affected by sgp130Fc.

## 3. Discussion

The current study evaluated the impact of IL-6 family cytokines on cellular and molecular responses in HUVECs and showed that gp130 signaling induces the release of a wide range of proteins involved in inflammatory responses, as well as cardiovascular physiology and pathophysiology. The present investigation also discloses differences and similarities between IL-6 family cytokines in their ability to induce intracellular signaling, regulate gene expression, and release inflammatory proteins from HUVECs.

In our study, we showed that five of the alpha receptors, and the common signal transducing receptor gp130, are expressed at the mRNA level in HUVECs. We observed that, among the alpha receptors, LIF-R and OSM-R are highly expressed, whereas IL-6R, IL-11R, and IL-27R showed lower levels of expression. The expression of gp130, on the other hand, is much higher than any of the studied alpha receptors. This has also been shown in other cell types known to respond to IL-6 family cytokines, such as hepatocytes [26]. We have shown previously that the expression of IL-6R and gp130 on the surface of endothelial cells is regulated by pro-inflammatory stimuli [15]. In accordance with this, we found, in the current study, that the transcription of most of the alpha receptors, and gp130, is regulated by TNF-α. While the expression of gp130, OSM-R, and IL-27R is upregulated, the expression of IL-6R and LIF-R is downregulated in endothelial cells exposed to TNF-α. Reduced IL-6R and enhanced gp130 expression on the surface of endothelial cells due to TNF-α may render endothelial cells more sensitive to IL-6 trans-signaling, rather than classic signaling. Hence, TNF-α mediated regulation of alpha receptors and gp130 might suggest an altered response of vascular endothelial cells to IL-6 family cytokines during inflammatory environments.

It is well known that endothelial cells can respond to different cytokines and release a wide range of biological mediators involved in a number of functions. In the current study, we performed a targeted proteomic analysis, and revealed that endothelial cell activation by IL-6 family cytokines regulated the release of several proteins that are involved in inflammatory responses and cardiovascular pathophysiology. Overall, OSM appeared to be the most potent in regulating the release of the proteins in question, of which some are solely induced by OSM. The other IL-6 family cytokines, LIF, CT-1, IL-6, and IL-11, also influence release of the same proteins regulated by OSM, but to a lesser degree. Further, we found that IL-6 and IL-11 trans-signaling induced the release of more proteins, and to a higher degree, than their classic-signaling counterparts. Although vascular endothelial cells express and release LIF [27], there are limited data regarding the impact of LIF on human endothelial responses [28]. This study reports that LIF induces the release of several cytokines, chemokines, and growth factors from HUVECs. Meanwhile, CNTF, CLC, and IL-27 had no or minimal effect on the release of proteins.

We found that the top three upregulated proteins induced by IL-6 family cytokines are CCL23, HGF, and IL-6. Gp130 signaling in endothelial cells has been previously shown to induce production of the pleiotropic cytokine IL-6 [2,29]. In addition, we have previously reported that CCL23, a chemokine predominantly formed by neutrophils [30,31,32] in response to TLR-agonists or TNF-α, can be regulated by IL-6 in endothelial cells in an autocrine manner [33]. However, to our knowledge, the current study is the first to demonstrate the role of IL-6 family cytokines in inducing CCL23 and HGF. Given that HGF, CCL23, and IL-6 are known to be involved in angiogenesis [14,34,35,36], the upregulation of these proteins in our study implies a role for IL-6 family cytokines in angiogenesis.

Furthermore, pathway analysis also indicated that IL-6 family cytokines regulate functions related to chemotaxis, recruitment, and invasion, suggesting a role of gp130 in immune cell recruitment to the vascular wall. This is in line with previous studies showing that IL-6 family cytokines induce the release of chemokines and expression of adhesion molecules [2,15,37,38], and is further supported by a previous study showing that mice deficient in endothelial gp130 display reduced trans-endothelial migration of neutrophils [39].

Indeed, further pathway analyses, using all differentially regulated proteins by IL-6 family cytokines, indicated that all enriched functions were predicted to be activated. We noted that OSM, LIF, and IL-6 trans-signaling appeared to enrich these functions more strongly than the other cytokines. Interestingly, there are functions that were distinctly regulated by OSM (e.g., activation of phagocytes) and IL-6 trans-signaling (e.g., organization and cell death). Nevertheless, further functional studies using *in vitro* or *in vivo* models and endothelial cells from other sources are required to further elucidate the impact of IL-6 family cytokines on endothelial functions. Overall, our findings reveal pleiotropic, shared, but also unique responses induced by IL-6 family cytokines in human vascular endothelial cells.

In line with the proteomic data, gene expression analyses of the top three upregulated proteins (CCL23, HGF, and IL-6) revealed that the magnitude of induction caused by different IL-6 family cytokines is also mirrored on the transcriptional level. This led us to further investigate the typical signaling pathways downstream of gp130, which included the JAK/STAT3, PI3-K/AKT, and MEK/ERK pathways [11,12,13]. Differences in signal strength and diversity among various IL-6 family cytokines, as well as the relative role played by these intracellular pathways in endothelial cells, is not well established. We have previously shown that IL-6 trans-signaling, compared to classic signaling, induces broader intracellular signaling in endothelial cells [15]. Specifically, we found that IL-6 classic and trans-signaling activated the JAK/STAT3 signaling pathway. On the other hand, only trans-signaling caused MEK/ERK and PI3-K/AKT activation. In the current study, we showed that STAT3 is phosphorylated in endothelial cells in response to OSM, LIF, CT-1, IL-6, and IL-11. We noted that OSM, IL-6 trans-signaling, and LIF resulted in the strongest signaling amplitudes. In addition, ERK1/2 phosphorylation was induced by OSM and IL-11 trans-signaling, while phosphorylation of AKT was seen in response to OSM and CT-1. Following a similar pattern, the gene expression of SOCS3, which is a known negative regulator of gp130 signaling [40], was upregulated by OSM, LIF, CT-1, IL-6 trans-signaling, IL-11 classic and trans-signaling, and CNTF, although OSM and IL6-trans-signaling caused the most pronounced increase.

Furthermore, we showed that IL-11, combined with its soluble receptor, induces phosphorylation of STAT3 and ERK1/2, whereas IL-11 alone had no or limited effects. This suggests that that the concepts of classic and trans-signaling that we and others have reported previously [15,41] also apply to IL-11 in human endothelial cells. The difference in intra-cellular signaling is also reflected in gene expression, as well as protein release, induced by both IL-11 and IL-6, where classic signaling induced minor non-significant induction, while trans-signaling resulted in pronounced increases in gene and protein expression. This may partly be due to the limited number of receptors available for classic signaling, while trans-signaling directly activates the much more abundant gp130 receptor, which enables it to significantly enhance the amplitude of downstream signaling, and hence an enhanced response [42]. It may also be due to differential phosphorylation of the intracellular domain of the gp130 receptor during classic versus trans-signaling, which subsequently dictates the engagement of the pathways to be activated.

Previous studies have reported that IL-6 trans-signaling and IL-11 trans-signaling can be antagonized by a naturally occurring soluble gp130 receptor (sgp130) [43,44]. Sgp130 is generated from membrane cleavage and alternative splicing [45], and is present in high concentrations in the circulation system [46]. In the current study, we showed that sgp130Fc effectively blocks IL-6 trans-signaling-induced responses in HUVECs, and reduces responses induced by IL-11-trans-signaling. However, there was no effect on responses induced by OSM, LIF, or CT-1. Previous data suggest that sgp130 has 10–1000 times higher affinity to the IL-11/sIL-11R and IL-6/sIL-6R complexes compared to OSM [47,48,49,50], which might explain why OSM signaling is not affected by sgp130 while IL-6 trans- and IL-11 trans-signaling-induced responses were significantly inhibited in our study. However, we cannot rule out the possibility that sgp130, at a much higher concentration, might interfere with OSM signaling. For instance, a previous study utilizing a 10 times higher concentration of sgp130 showed some degree of OSM-signaling inhibition [51]. Taken together, this suggests that sgp130 preferentially blocks trans-signaling-induced responses but does not affect responses induced by classic signaling in human vascular endothelial cells.

Our study revealed both qualitative and quantitative differences in intra-cellular signaling pathways, in addition to differentially regulated proteins and functions, by IL-6 family cytokines. For instance, OSM induced the most prominent activation of intracellular signaling, and was the most efficient in regulating gene expression and the release of proteins. This may be due to a high expression of OSM-receptors on endothelial cells, [52] but may also be related to the signaling capability of OSM, which can utilize both the OSM receptor and the LIF-receptor [24,53]. Furthermore, it has been proposed that gp130 possesses unique binding epitopes for different cytokines/cytokine receptors [54,55], which might allow an opportunity for differences in subsequent intracellular signaling and further cellular effects induced by the different IL-6 family cytokines. The limited or lack of response to CNTF, CLC, and IL-27 could be attributed to limited or lack of receptor expression. Particularly, IL-27 did not induce phosphorylation of STAT3, AKT, or ERK1/2, and did not affect the transcription or release of any of the measured proteins. This contrasts with previous findings showing that IL-27 induced phosphorylation of STAT3, as well as increased expression of CXCL11 and IL-6, among others, in human endothelial cells [3,38]. These discrepancies may be related to differences in cytokine concentration, but may also be a consequence of different cellular origin, or post-transcriptional modifications of the cytokine.

## 4. Materials and Methods

### 4.1. Cell Culture

Human umbilical vein endothelial cells (HUVECs) (Thermo Fisher Scientific, Waltham, MA, USA) were cultured in complete endothelial medium (VascuLife basal medium supplemented with VEGF LifeFactors kit (LifeLine Cell Technologies, Frederick, MD, USA)), including antibiotics (Penicillin (0.1 U/mL) + Streptomycin (100 ng/mL) (Thermo Fisher Scientific, Waltham, MA, USA). The cultures were kept at 37 °C and 5% CO_2_ environment, and cells were used in passage 4–9. The medium was replaced every 48–72 h, and sub-culturing was made upon confluence. For analysis of gene expression or intracellular signaling pathways, 3 × 10^5^ cells/well were seeded in 6-well plates (Sarstedt, Nümbrecht, Germany). For measurement of released proteins, 6 × 10^4^ cells/well were seeded in 24-well plates (Sarstedt, Nümbrecht, Germany). HUVECs were seeded in complete endothelial medium containing antibiotics. The next day, the medium was removed from wells with confluent monolayers of HUVECs and replaced with fresh complete endothelial medium, without antibiotics, and cells were treated with OSM, LIF, CT-1, CLC, CNTF, IL-27, IL-11, sIL-11R, IL-6, sIL-6R, or soluble gp130 (sgp130Fc) obtained from Bio-techne (Minneapolis, MN, USA). Dose–response experiments were performed to establish the concentration of cytokines that should be used in subsequent experiments. Time of treatment ranged from 2 min to 48 h. At the end of each treatment, cell culture medium and/or cells were collected and kept at −80 °C until further analysis.

### 4.2. Proteomics

To analyze the release of a wide range of proteins by HUVECs in response to IL-6 family cytokines, proteomic analyses were performed using Proximity Extension Assay (PEA) by Olink^®^ Proteomics (Uppsala Sweden). Cell culture medium was collected 48 h after exposure to 10 µg/mL of OSM, LIF, CT-1, CLC, CNTF, IL-27, IL-11, sIL-11R, IL-6, or sIL-6R; 92 proteins in the Cardiovascular III panel, and 92 proteins in the Inflammation panel, were measured. The amount of released protein was compared to untreated cells and reported as the fold change (FC) of normalized protein expression (NPX) on a log2 scale.

### 4.3. RNA Isolation and cDNA Synthesis

RNA was isolated from HUVECs after 2, 8, and 24 h of cytokine treatment, in the presence or absence of 1 µg/mL sgp130Fc, using an E.Z.N.A.^®^ Total RNA Kit I (Omega Bio-tek, Norcross, GA, USA), according to the manufacturer’s instructions. The concentration of RNA in the samples was determined using Nano-drop 2000 (Thermo Fisher Scientific, Waltham, MA, USA). cDNA was synthesized using a high-capacity cDNA reverse transcription kit (Applied Biosystems, Thermo Fisher Scientific, Waltham, MA, USA), according to the manufacturer’s instructions. cDNA synthesis was performed in 20 µL reactions containing 1 µg RNA, using the following thermal program: 10 min at 25 °C, 120 min at 37 °C, and 5 min at 85 °C. A negative control sample, which contained water instead of RNA, was included. All samples were cooled down to 4 °C and stored at −20 °C until analysis.

### 4.4. Quantitative Real-Time PCR

Gene expression analyses were performed using quantitative real-time PCR (qPCR) in 10 µL reactions containing LuminoCt^®^ qPCR ReadyMix™ (Sigma-Aldrich, St. Louise, MO, USA), Taqman primers/probes (Applied Biosystems, Thermo Fisher Scientific, Waltham, MA, USA), cDNA, and water. The following cycling conditions were used: initialization at 95 °C for 20 s, followed by 40 cycles of 95 °C for 1s, and 60 °C for 20 s in a QuantStudio 7 Flex Realtime PCR system (Applied Biosystems, Thermo Fisher Scientific, Waltham, MA, USA). A six-point standard curve was included, which was prepared by making 1:2 dilutions of pooled cDNA. Gene expression data was normalized to the expression of GAPDH. Specific Taqman primers/probes used are listed in Supplementary Materials.

### 4.5. Western Blotting

Western blotting was used to evaluate the signaling protein phosphorylation induced by the IL-6 family cytokines, using cell lysates of HUVECs treated with cytokines for 2–60 min. Cells were lysed with ice-cold RIPA-buffer (Merck Millipore, Burlington, MA, USA) containing Halt™ Phosphatase Inhibitor Cocktail (Thermo Fisher Scientific, Waltham, MA, USA). Proteins were quantified with a Micro BCA™ Protein Assay kit (Thermo Fisher Scientific, Waltham, MA, USA), according to the manufacturer’s instructions, and absorbance was measured at 540 nm using a Cytation 3 imaging reader (BioTek, Winooski, VT, USA). Cell lysates were mixed with sodium dodecyl sulfate (SDS, Sigma-Aldrich) and denatured for 5 min at 95 °C. Proteins (10–20 µg) were loaded into NuPAGE™ 4–12% Bis-Tris Gels (Thermo Fisher Scientific, Waltham, MA, USA). Protein separation was performed at 140 V in NuPAGE^®^ MOPS SDS Running Buffer (Thermo Fisher Scientific, Waltham, MA, USA). Magic Mark™ XP and Novex^®^ Sharp Pre-Stained protein standards (both from Thermo Fisher Scientific, Waltham, MA, USA) were used to determine molecular weight. Proteins were blotted on Immobilion^®^ FL Transfer Membrane (Merck Millipore, Burlington, MA, USA) and blocked with 5% bovine serum albumin in TBS-T (10 mM Tris-HCl pH 8.0, 150 mM NaCl, 0.1% (*v*/*v*) Tween-20). Membranes were probed with phospho-STAT3 (Tyr705) antibody (rabbit polyclonal #9131, Cell Signaling Technology, Danvers, MA, USA, diluted 1:1000), phospho-AKT (Ser473) antibody (rabbit monoclonal, #4060, Cell signaling Technology, Danvers, MA, USA, diluted 1:1000), phospho-ERK1/2 antibody (Thr202/Tyr204) (mouse monoclonal, #9106, Cell Signaling Technology, Danvers, MA, USA, diluted 1:1000), and β-Tubulin antibody (mouse monoclonal, #05–661, Merck Millipore, Burlington, MA, USA, diluted 1:2000). Membranes were rinsed in TBS-T before incubation with horseradish peroxidase (HRP)-conjugated goat anti-rabbit IgG (#7074, Cell Signaling Technology, diluted 1:2000) or horse anti-mouse IgG (#7074, Cell Signaling Technology, Danvers, MA, USA, diluted 1:2000). Proteins were visualized using Immobilon™ Western Chemiluminescent HRP Substrate solution (Merck Millipore, Burlington, MA, USA), and chemiluminescence was detected by Li-Cor Odyssey Fc imager and analyzed with Image Studio Software (LI-COR Biosciences, Lincoln, NE, USA). Protein expression data were normalized to the expression of β-tubulin and the control was set to 1.

### 4.6. Statistical Analysis

Gene expression analysis and protein release, determined by ELISA, was analyzed using one-way ANOVA followed by Dunnett’s multiple comparison test, using GraphPad Prism 6. For proteins detected with PEA, *t*-test were performed, followed by the Benjamini–Hochberg correction for multiple comparisons.

### 4.7. Ingenuity Pathway Analysis

Released proteins, determined by PEA, were further evaluated using the Ingenuity pathway (IPA^®^, QIAGEN Inc., The Netherlands) online tool [56] to identify pathways and biological functions regulated by gp130-signaling cytokines. Core analyses were performed using a fold change cutoff of 1.5 (0.5850 on log2 scale), *p*-value of 0.05, and false discovery rate of 0.2. Enriched biological functions were identified using a z-score cutoff > 2. IPA analysis was performed on 7 October 2020 (https://www.qiagenbioinformatics.com/products/ingenuity-pathway-analysis).

## 5. Conclusions

This study reports the differences and similarities between molecular and cellular responses induced by IL-6 family cytokines in human endothelial cells. The release and transcription of proteins induced by OSM, LIF, CT-1, IL-6, and IL-11 (classic and trans-signaling), follow a very similar pattern, while CNTF, CLC, and IL-27 had no or limited effect. This study also identified proteins involved in angiogenesis and immune cell recruitment, which are regulated by IL-6 family cytokines. Furthermore, this study shows that sgp130 effectively blocks trans-signaling-induced responses by IL-6 and IL-11 in endothelial cells but does not affect responses mediated via membrane-bound receptors.

## Figures and Tables

**Table 1:** Protein release induced by IL-6 family cytokines in human umbilical vein endothelial cells.

Fold Change (log2) Compared to Untreated Cells								
	OSM	LIF	CT-1	IL-6 + sIL-6R	IL-11 + sIL-11R	IL-6	IL-11	CNTF
**CCL23**	**5.04**	**2.29**	2.47	**4.17**	2.56	1.83	1.61	0.77
**HGF**	**4.75**	**3.23**	**3.41**	**3.22**	2.92	1.88	2.03	**1.05**
**IL-6**	**4.72**	**2.12**	2.37	-	**2.66**	-	1.38	0.49
**TR-AP**	**2.93**	1.48	1.41	**2.90**	1.68	0.96	0.54	0.16
**MCP-1**	**2.55**	1.00	1.08	**2.47**	1.69	0.74	0.50	−0.01
**CXCL5**	**1.88**	0.74	0.92	1.81	0.80	0.73	0.79	0.18
**MCP-3**	**1.77**	**1.22**	1.40	**1.72**	1.33	0.72	1.21	0.4
**TRAIL**	**1.56**	**0.84**	1.05	1.46	0.81	0.66	0.91	0.31
**OPG**	1.34	**0.88**	0.92	1.09	0.68	0.51	1.09	0.32
**CSF-1**	**0.94**	**1.09**	1.17	0.92	0.92	0.47	1.19	0.46
**TWEAK**	0.91	**0.69**	**0.80**	0.81	0.62	0.48	1.08	0.32
**IL-18R1**	0.58	**0.63**	0.74	0.61	0.45	0.4	0.93	0.20
**IL-15RA**	−0.03	0.13	0.33	**1.72**	0.16	0.09	0.30	0.06
**JAM-A**	−0.31	0.05	−0.02	**0.73**	0.01	0.26	0.08	−0.27
**4E-BP1**	−**0.69**	−0.19	0	−0.44	0.16	−0.99	0.55	−0.53
**CASP-3**	−**0.86**	−0.28	−0.27	0.08	−0.02	−0.35	0.09	−0.56
**ADA**	−**0.93**	−0.14	−0.12	−0.37	−0.22	−0.44	0.43	−0.38
**CCL20**	−**1.42**	−0.80	−0.53	−**0.37**	−0.75	−0.20	0.31	0.07

Release of proteins from human umbilical vein endothelial cells was analyzed by proximity extension assay (Olink^®^ Proteomics) using the Cardiovascular III and Inflammation panels. Data shown are fold change (log2) compared to untreated controls. Proteins with a log2 fold change >0.58 or <−0.58 and Benjamini–Hochberg false discovery rate (FDR) < 0.1 in any of the treatments are shown. Log2 fold change indicated in bold represents FDR < 0.1. Upregulated proteins are indicated in blue, and downregulated proteins indicated in orange. Light colors represent log2 fold change >0.58, and dark colors represent log2 fold change >1. Abbreviations: oncostatin M (OSM), leukemia inhibitory factor (LIF), cardiotrophin 1 (CT-1), interleukin (IL), ciliary neurotrophic factor (CNTF), C-C motif chemokine 23 (CCL23), hepatocyte growth factor (HGF), tartrate-resistant acid phosphatase type 5 (TR-AP), monocyte chemotactic protein 1 (MCP-1), C-X-C motif chemokine 5 (CXCL5), monocyte chemotactic protein 3 (MCP-3), TNF-related apoptosis-inducing ligand (TRAIL), osteoprotegerin (OPG), macrophage colony-stimulating factor 1 (CSF-1), tumor necrosis factor (Ligand) superfamily member 12 (TWEAK), interleukin-18 receptor 1 (IL-18R1), interleukin-15 receptor subunit alpha (IL-15RA), junctional adhesion molecule A (JAM-A), eukaryotic translation initiation factor 4E-binding protein 1 (4E-BP1), caspase-3 (CASP-3), adenosine deaminase (ADA), and C-C motif chemokine 20 (CCL20).

## Data Availability

Not applicable.

## Supplementary Materials

The following are available online at https://www.mdpi.com/article/10.3390/ijms23031448/s1.
