# Supplementary material for: Pleiotropic, Unique and Shared Responses Elicited by IL-6 Family Cytokines in Human Vascular Endothelial Cells
Source: Int J Mol Sci. 2022 Jan 27;23(3):1448. doi: 10.3390/ijms23031448 (PMC8836206; doi:10.3390/ijms23031448)
Supplement: Supplementary file 1 [file ijms-23-01448-s001.zip › ijms-1541039-supplementary.pdf]

Supplementary Materials

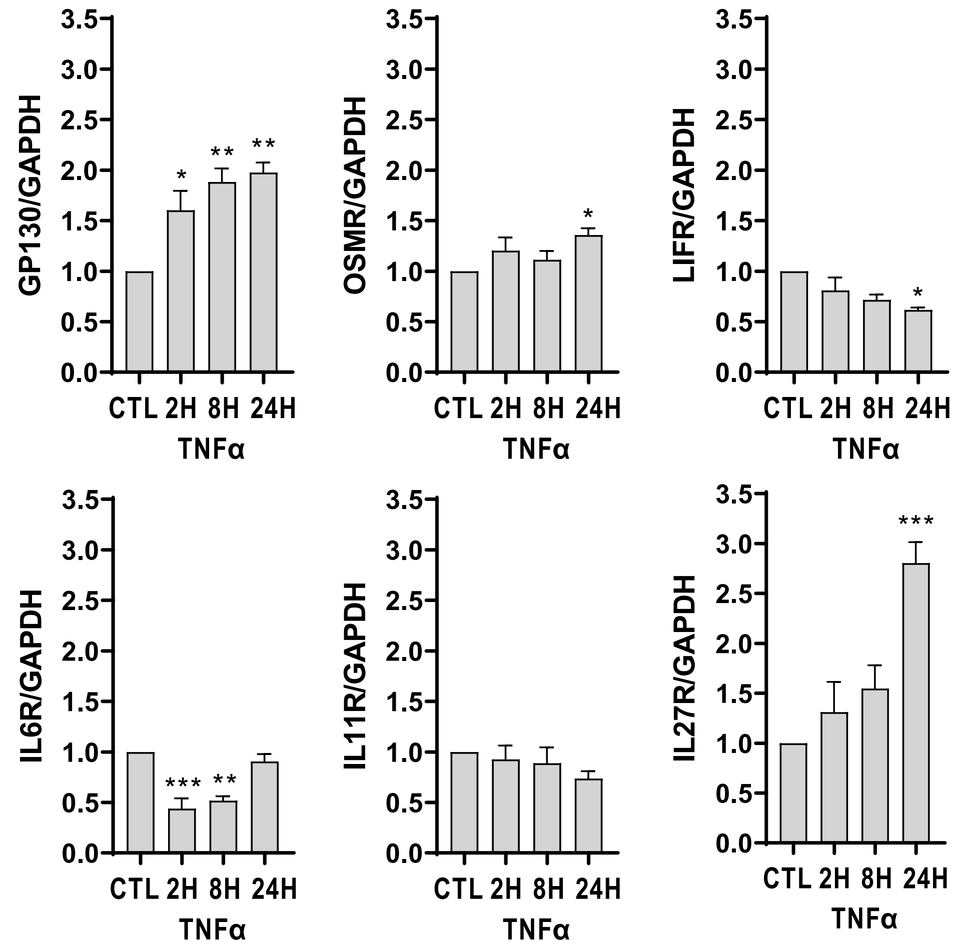

Supplementary Figure S1. Gene expression of IL6-family cytokine receptors in human endothelial cells after exposure to tumor necrosis factor alpha (TNFα). Human endothelial cells were exposed to TNFα 10ng/ml for 2-24 hours. Gene expression was analysed using Taqman quantitative PCR. Data was normalized to GAPDH, and untreated controls were set to 1. Data shown are mean ± SEM from 3 independent experiments run in duplicate. Statistical significance was calculated using One-way ANOVA followed by Dunnet´s post hoc test. * $p < 0.05$, ** $p < 0.01$, *** $p < 0.001$.

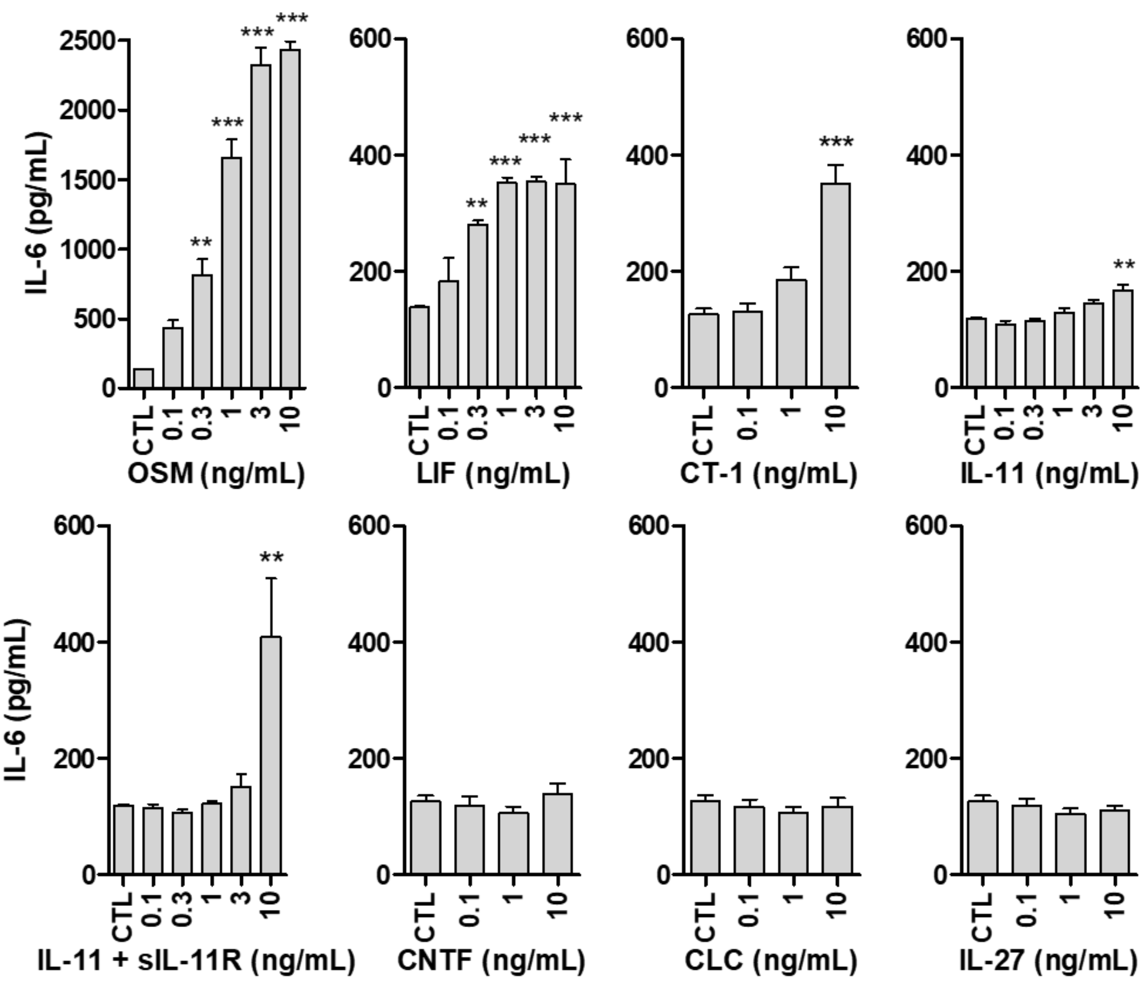

Supplementary Figure S2. Dose-response relationship of IL-6 family cytokines and release of interleukin-6 from human endothelial cells. Human endothelial cells were exposed to oncostatin M (OSM), leukemia inhibitory factor (LIF), cardiotrophin 1 (CT-1), interleukin-11 alone (IL-11) and in combination with soluble IL11-receptor (IL11 + R), ciliary neurotrophic factor (CNTF), cardiotrophin-like cytokine (CLC) or interleukin 27 for 48h. Release of IL6 was measured in cell supernatant using ELISA. Data shown are mean ± SEM from 3 independent experiments run in duplicate. Statistical significance was calculated using One-way ANOVA followed by Dunnet´s post hoc test. * $p < 0.05$, ** $p < 0.01$, *** $p < 0.001$.

Materials and Methods

ELISA

Enzyme-linked immunosorbent assay (ELISA, DuoSet®, Bio-techne) was used to measure the release of IL-6 as a response to increasing concentration of OSM, LIF, CT-1, CLC, CNTF, IL-27, IL-11, sIL-11R. The analysis was performed according to the manufacturer's instructions. Absorbance was measured at 450nm using Cytation 3 imaging reader (BioTek, Winooski, VT, USA).

Taqman primers/probes

Taqman primer/probes	
CCL23	Hs00270756_m1
CNTFR	Hs00181798_m1
GAPDH	Hs99999905_m1
HGF	Hs00300159_m1
IL11RA	Hs00234415_m1
IL27RA	Hs00945029_m1
IL6	Hs00174131_m1
IL6R	Hs01075664_m1
IL6ST (gp130)	Hs00174360_m1
LIFR	Hs01123581_m1
OSMR	Hs00384276_m1
SOCS3	Hs02330328_m1
STAT3	Hs00374280_m1

Supplementary Table S1

	Uniprot ID	OSM				LIF				CT-1				IL-6 + sIL6R				IL-11 + sIL11R			
		FC	SD	p	FDR	FC	SD	p	FDR	FC	SD	p	FDR	FC	SD	p	FDR	FC	SD	p	FDR
**CCL23**	P55773	5.04	0.43	0.004	**0.062**	2.29	0.15	0.002	**0.051**	2.47	0.39	0.012	0.171	4.17	0.57	0.009	**0.065**	2.56	0.24	0.004	0.147
**HGF**	P14210	4.75	0.32	0.002	**0.052**	3.23	0.20	0.002	**0.051**	3.41	0.25	0.003	**0.095**	3.22	0.19	0.002	**0.025**	2.92	0.33	0.006	0.147
**IL6**	P05231	4.72	0.24	0.001	**0.045**	2.12	0.24	0.006	**0.054**	2.37	0.34	0.010	0.171	-	-	-	-	2.66	0.13	0.001	**0.084**
**TR-AP**	P13686	2.93	0.41	0.010	**0.087**	1.48	0.43	0.04	0.123	1.41	0.37	0.032	0.188	2.90	0.55	0.017	**0.094**	1.68	0.32	0.018	0.230
**MCP-1**	P13500	2.55	0.41	0.013	**0.087**	1.00	0.38	0.065	0.151	1.08	0.49	0.090	0.292	2.47	0.34	0.009	**0.065**	1.69	0.34	0.02	0.230
**CXCL11**	O14625	1.96	0.54	0.035	0.130	1.34	0.43	0.047	0.123	1.54	0.37	0.028	0.185	1.45	0.46	0.048	0.136	1.07	0.60	0.129	0.325
**CXCL5**	P42830	1.88	0.38	0.020	0.100	0.74	0.23	0.046	0.123	0.92	0.46	0.105	0.304	1.81	0.42	0.026	0.124	0.8	0.38	0.095	0.325
**MCP-3**	P80098	1.77	0.32	0.016	**0.087**	1.22	0.20	0.014	**0.079**	1.40	0.33	0.027	0.185	1.72	0.22	0.008	**0.065**	1.33	0.35	0.033	0.298
**MCP-2**	P80075	1.64	0.43	0.033	0.129	0.87	0.45	0.114	0.215	0.83	0.45	0.120	0.304	1.19	0.47	0.070	0.160	0.61	0.40	0.165	0.377
**TRAIL**	P50591	1.56	0.24	0.011	**0.087**	0.84	0.09	0.005	**0.054**	1.05	0.24	0.026	0.185	1.46	0.36	0.029	0.124	0.81	0.26	0.047	0.298
**OPG**	O00300	1.34	0.47	0.058	0.171	0.88	0.13	0.01	**0.075**	0.92	0.23	0.029	0.185	1.09	0.33	0.043	0.136	0.68	0.32	0.099	0.325
**CSF-1**	P09603	0.94	0.20	0.021	0.100	1.09	0.18	0.013	**0.079**	1.17	0.18	0.012	0.171	0.92	0.25	0.036	0.136	0.92	0.34	0.062	0.325
**TWEAK**	O43508	0.91	0.36	0.069	0.171	0.69	0.13	0.016	**0.089**	0.80	0.04	0.002	**0.095**	0.81	0.42	0.114	0.195	0.62	0.32	0.114	0.325
**IGFBP-1**	P08833	0.66	0.17	0.032	0.129	0.37	0.14	0.063	0.151	0.35	0.19	0.121	0.304	1.30	0.32	0.03	0.124	0.48	0.15	0.045	0.298
**CCL15**	Q16663	0.60	0.15	0.028	0.123	0.23	0.13	0.138	0.242	0.10	0.20	0.539	0.648	1.13	0.44	0.068	0.160	0.26	0.14	0.118	0.325
**IL-18R1**	Q13478	0.58	0.32	0.122	0.249	0.63	0.12	0.019	**0.091**	0.74	0.23	0.043	0.233	0.61	0.30	0.102	0.193	0.45	0.30	0.171	0.377
**CXCL16**	Q9H2A7	0.51	0.05	0.005	**0.067**	0.28	0.06	0.02	**0.091**	0.27	0.12	0.085	0.292	1.01	0.32	0.047	0.136	0.34	0.09	0.036	0.298
**CXCL6**	P80162	0.50	0.27	0.119	0.249	0.60	0.44	0.194	0.309	0.77	0.66	0.238	0.462	0.56	0.38	0.174	0.249	0.31	0.83	0.65	0.785
**IGFBP-7**	Q16270	0.48	0.32	0.163	0.292	0.59	0.15	0.03	0.118	0.54	0.19	0.054	0.250	1.59	0.40	0.03	0.124	0.78	0.24	0.046	0.298
**CDCP1**	Q9H5V8	0.44	0.44	0.293	0.428	0.54	0.05	0.004	**0.054**	0.67	0.15	0.024	0.185	0.32	0.06	0.017	**0.094**	0.39	0.22	0.127	0.325
**Flt3L**	P49771	0.42	0.40	0.281	0.425	0.42	0.11	0.033	0.121	0.51	0.23	0.092	0.292	0.53	0.23	0.081	0.163	0.38	0.13	0.058	0.325
**TFPI**	P10646	0.40	0.26	0.161	0.292	0.51	0.11	0.022	**0.095**	0.50	0.21	0.081	0.292	1.12	0.38	0.053	0.144	0.67	0.28	0.077	0.325
**IL-18BP**	O95998	0.35	0.14	0.072	0.171	0.13	0.16	0.389	0.484	0.21	0.18	0.239	0.462	0.76	0.30	0.069	0.160	0.29	0.05	0.017	0.230
**CXCL1**	P09341	0.29	0.47	0.478	0.587	0.37	0.15	0.072	0.157	0.27	0.21	0.212	0.436	0.43	0.26	0.149	0.232	0.2	0.28	0.430	0.636
**TNF-R1**	P19438	0.24	0.32	0.396	0.504	0.51	0.08	0.011	**0.075**	0.34	0.43	0.377	0.535	0.80	0.26	0.049	0.136	0.57	0.29	0.108	0.325
**GRN**	P28799	0.24	0.24	0.285	0.425	0.39	0.04	0.006	**0.054**	0.32	0.17	0.115	0.304	0.90	0.26	0.041	0.136	0.47	0.22	0.092	0.325
**SELP**	P16109	0.16	0.19	0.367	0.501	0.26	0.21	0.213	0.324	0.13	0.28	0.586	0.684	0.63	0.44	0.181	0.253	0.27	0.14	0.106	0.325
**GDF-15**	Q99988	0.13	0.31	0.626	0.718	0.47	0.14	0.042	0.123	0.44	0.22	0.106	0.304	0.90	0.29	0.048	0.136	0.58	0.30	0.115	0.325
**LTBR**	P36941	0.08	0.10	0.372	0.501	0.10	0.14	0.401	0.484	0.25	0.13	0.120	0.304	0.58	0.09	0.011	**0.068**	0.18	0.09	0.106	0.325
**MMP-1**	P03956	0.05	0.25	0.798	0.846	-0.32	0.03	0.003	**0.054**	-0.19	0.08	0.083	0.292	0.11	0.22	0.557	0.629	-0.01	0.10	0.904	0.958
**EPHB4**	P54760	0.02	0.01	0.197	0.328	0.25	0.07	0.036	0.123	0.17	0.11	0.147	0.322	0.58	0.32	0.125	0.208	0.17	0.13	0.205	0.395
**ST2**	Q01638	0.01	0.48	0.969	0.969	0.40	0.27	0.167	0.279	0.33	0.14	0.080	0.292	0.73	0.35	0.099	0.192	0.45	0.18	0.073	0.325
**TRANCE**	O14788	-0.02	0.16	0.879	0.905	0.41	0.16	0.071	0.157	0.29	0.34	0.349	0.520	-0.43	0.03	0.002	**0.025**	0.21	0.47	0.593	0.740
**IL-15RA**	Q13261	-0.03	0.32	0.900	0.913	0.13	0.16	0.369	0.470	0.33	0.11	0.049	0.245	1.72	0.18	0.005	**0.054**	0.16	0.29	0.506	0.694
**IL-10RB**	Q08334	-0.07	0.24	0.738	0.794	0.20	0.31	0.465	0.525	0.24	0.20	0.244	0.462	0.02	0.07	0.736	0.780	0.1	0.17	0.481	0.674
**CD93**	Q9NPY3	-0.07	0.24	0.719	0.787	0.31	0.01	0.001	**0.049**	0.23	0.23	0.300	0.488	0.62	0.27	0.081	0.163	0.37	0.24	0.159	0.377
**PLC**	P98160	-0.08	0.29	0.719	0.787	0.34	0.11	0.053	0.133	0.25	0.25	0.281	0.488	0.41	0.27	0.170	0.247	0.34	0.23	0.175	0.377
**LAP TGF-beta-1**	P01137	-0.09	0.54	0.836	0.874	0.67	0.21	0.045	0.123	0.61	0.13	0.023	0.185	0.06	0.36	0.845	0.858	0.13	0.56	0.777	0.892
**SCF**	P21583	-0.10	0.20	0.563	0.657	0.15	0.08	0.12	0.222	0.27	0.21	0.212	0.436	0.02	0.12	0.829	0.853	0.04	0.23	0.830	0.907
**CTSZ**	Q9UBR2	-0.10	0.16	0.461	0.576	0.14	0.04	0.041	0.123	0.10	0.15	0.468	0.589	0.45	0.24	0.114	0.195	0.17	0.13	0.213	0.395
**ICAM-2**	P13598	-0.11	0.32	0.682	0.770	0.24	0.14	0.136	0.242	0.15	0.25	0.479	0.589	0.59	0.24	0.073	0.160	0.18	0.21	0.356	0.567
**MPO**	P05164	-0.11	0.14	0.392	0.504	-0.14	0.10	0.185	0.302	-0.08	0.16	0.546	0.648	-0.05	0.06	0.336	0.399	-0.04	0.10	0.603	0.740

	IL-6				IL-11				CNTF				CLC				IL-27			
	FC	SD	p	FDR	FC	SD	p	FDR	FC	SD	p	FDR	FC	SD	p	FDR	FC	SD	p	FDR
**CCL23**	1.83	0.57	0.046	0.460	1.61	0.38	0.026	0.252	0.77	0.18	0.030	0.220	-0.20	0.71	0.724	0.853	0.24	0.42	0.502	0.996
**HGF**	1.88	0.34	0.016	0.271	2.03	0.31	0.011	0.252	1.05	0.04	0.001	**0.050**	-0.22	0.43	0.549	0.853	0.14	0.50	0.736	0.996
**IL6**	-	-	-	-	1.38	0.45	0.050	0.252	0.49	0.15	0.045	0.241	-0.15	0.14	0.278	0.853	0.01	0.61	0.985	0.996
**TR-AP**	0.96	0.73	0.204	0.512	0.54	0.32	0.140	0.327	0.16	0.08	0.095	0.335	-0.41	0.56	0.408	0.853	-0.16	0.18	0.335	0.996
**MCP-1**	0.74	0.58	0.211	0.512	0.50	0.29	0.134	0.327	-0.01	0.33	0.957	0.981	-0.23	0.52	0.596	0.853	-0.13	0.44	0.711	0.996
**CXCL11**	0.97	0.15	0.012	0.271	1.43	0.10	0.003	0.176	0.83	0.39	0.094	0.335	0.10	0.56	0.834	0.871	0.58	0.36	0.148	0.996
**CXCL5**	0.73	0.16	0.022	0.271	0.79	0.37	0.096	0.308	0.18	0.15	0.236	0.636	-0.09	0.43	0.784	0.853	0.28	0.24	0.237	0.996
**MCP-3**	0.72	0.16	0.023	0.271	1.21	0.33	0.035	0.252	0.40	0.06	0.012	0.166	-0.14	0.30	0.58	0.853	-0.02	0.47	0.948	0.996
**MCP-2**	0.39	0.30	0.206	0.512	0.67	0.27	0.073	0.299	0.22	0.09	0.078	0.335	-0.12	0.40	0.72	0.853	0.19	0.27	0.427	0.996
**TRAIL**	0.66	0.36	0.122	0.512	0.91	0.32	0.058	0.253	0.31	0.10	0.045	0.241	-0.23	0.48	0.568	0.853	0.19	0.27	0.437	0.996
**OPG**	0.51	0.25	0.103	0.512	1.09	0.31	0.038	0.252	0.32	0.08	0.029	0.215	-0.23	0.36	0.466	0.853	0.13	0.53	0.769	0.996
**CSF-1**	0.47	0.30	0.158	0.512	1.19	0.25	0.021	0.252	0.46	0.06	0.008	0.148	-0.20	0.36	0.506	0.853	0.18	0.45	0.625	0.996
**TWEAK**	0.48	0.40	0.231	0.512	1.08	0.37	0.054	0.252	0.32	0.14	0.087	0.335	-0.17	0.46	0.647	0.853	0.08	0.65	0.874	0.996
**IGFBP-1**	0.46	0.30	0.160	0.512	0.33	0.20	0.145	0.327	0.05	0.35	0.857	0.981	-0.17	0.35	0.569	0.853	-0.06	0.26	0.788	0.996
**CCL15**	0.37	0.06	0.012	0.271	0.08	0.12	0.474	0.593	-0.01	0.05	0.760	0.981	-0.09	0.06	0.176	0.853	-0.07	0.05	0.226	0.996
**IL-18R1**	0.4	0.27	0.165	0.512	0.93	0.31	0.052	0.252	0.20	0.12	0.140	0.446	-0.22	0.17	0.202	0.853	0.02	0.54	0.971	0.996
**CXCL16**	0.4	0.29	0.197	0.512	0.25	0.12	0.100	0.308	0.12	0.38	0.711	0.981	-0.18	0.48	0.641	0.853	-0.02	0.28	0.940	0.996
**CXCL6**	0.33	0.16	0.096	0.512	1.04	0.51	0.101	0.308	0.23	0.17	0.193	0.563	0.08	0.37	0.792	0.853	0.44	0.26	0.137	0.996
**IGFBP-7**	0.74	0.57	0.209	0.512	0.55	0.56	0.300	0.482	0.09	0.56	0.842	0.981	-0.35	0.83	0.612	0.853	-0.08	0.62	0.871	0.996
**CDCP1**	0.25	0.16	0.155	0.512	0.90	0.42	0.096	0.308	0.24	0.24	0.289	0.750	-0.15	0.19	0.37	0.853	0.06	0.37	0.848	0.996
**Flt3L**	0.29	0.21	0.185	0.512	0.93	0.49	0.116	0.327	0.42	0.11	0.031	0.215	-0.11	0.30	0.67	0.853	0.16	0.62	0.752	0.996
**TFPI**	0.56	0.44	0.209	0.512	0.46	0.41	0.254	0.479	0.09	0.44	0.800	0.981	-0.35	0.76	0.578	0.853	0.01	0.49	0.975	0.996
**IL-18BP**	0.35	0.25	0.191	0.512	0.05	0.05	0.276	0.482	-0.13	0.31	0.622	0.981	-0.17	0.36	0.563	0.853	-0.11	0.25	0.606	0.996
**CXCL1**	0.15	0.13	0.234	0.512	0.67	0.29	0.085	0.308	0.08	0.01	0.014	0.166	-0.06	0.20	0.735	0.853	0.08	0.30	0.747	0.996
**TNF-R1**	0.54	0.44	0.222	0.512	0.43	0.36	0.228	0.47	0.07	0.48	0.850	0.981	-0.41	0.69	0.489	0.853	-0.29	0.51	0.501	0.996
**GRN**	0.42	0.39	0.265	0.512	0.35	0.35	0.294	0.482	0.05	0.35	0.847	0.981	-0.27	0.54	0.556	0.853	-0.01	0.43	0.987	0.996
**SELP**	0.31	0.27	0.248	0.512	0.11	0.13	0.343	0.509	0.01	0.05	0.789	0.981	-0.08	0.23	0.679	0.853	-0.08	0.09	0.33	0.996
**GDF-15**	0.58	0.40	0.177	0.512	0.48	0.44	0.260	0.479	0.22	0.47	0.566	0.981	-0.24	0.67	0.664	0.853	-0.04	0.52	0.929	0.996
**LTBR**	0.22	0.33	0.449	0.605	0.12	0.32	0.644	0.716	0.15	0.08	0.124	0.415	-0.06	0.18	0.686	0.853	0.03	0.18	0.839	0.996
**MMP-1**	0.02	0.05	0.621	0.724	-0.42	0.24	0.131	0.327	-0.14	0.26	0.538	0.981	0.17	0.10	0.152	0.853	0.04	0.32	0.860	0.996
**EPHB4**	0.21	0.21	0.291	0.512	0.25	0.25	0.303	0.482	-0.16	0.31	0.551	0.981	-0.43	0.59	0.409	0.853	-0.08	0.18	0.572	0.996
**ST2**	0.41	0.47	0.343	0.523	0.45	0.55	0.364	0.509	0.01	0.31	0.978	0.981	-0.28	0.48	0.490	0.853	-0.06	0.40	0.859	0.996
**TRANCE**	-0.07	0.32	0.777	0.837	0.73	0.11	0.010	0.252	0.18	0.12	0.167	0.507	-0.09	0.26	0.659	0.853	0.19	0.34	0.503	0.996
**IL-15RA**	0.09	0.27	0.685	0.779	0.30	0.13	0.082	0.308	0.06	0.16	0.669	0.981	-0.01	0.08	0.897	0.91	0.08	0.24	0.689	0.996
**IL-10RB**	0.12	0.19	0.479	0.633	0.42	0.12	0.038	0.252	0.13	0.38	0.671	0.981	-0.01	0.13	0.887	0.91	0.07	0.21	0.679	0.996
**CD93**	0.28	0.47	0.49	0.635	0.35	0.42	0.363	0.509	-0.03	0.33	0.914	0.981	-0.36	0.55	0.453	0.853	-0.07	0.49	0.855	0.996
**PLC**	0.25	0.31	0.373	0.544	0.31	0.34	0.327	0.498	0.04	0.25	0.863	0.981	-0.31	0.51	0.477	0.853	0.05	0.39	0.875	0.996
**LAP TGF-beta-1**	0.33	0.24	0.192	0.512	1.16	0.29	0.029	0.252	0.34	0.16	0.096	0.335	-0.14	0.37	0.655	0.853	0.24	0.44	0.525	0.996
**SCF**	0.13	0.14	0.323	0.513	0.61	0.34	0.130	0.327	0.11	0.16	0.436	0.954	0.04	0.06	0.467	0.853	0.09	0.34	0.747	0.996
**CTSZ**	0.29	0.30	0.30	0.512	0.29	0.25	0.247	0.479	0.03	0.31	0.915	0.981	-0.16	0.45	0.663	0.853	-0.02	0.34	0.943	0.996
**ICAM-2**	0.39	0.38	0.284	0.512	0.31	0.39	0.373	0.512	-0.01	0.37	0.981	0.981	-0.33	0.56	0.491	0.853	-0.06	0.33	0.812	0.996
**MPO**	-0.09	0.10	0.344	0.523	-0.05	0.14	0.632	0.714	-0.04	0.13	0.694	0.981	-0.08	0.17	0.576	0.853	0.02	0.12	0.867	0.996

Supplementary Table S1 (continued)

	Uniprot ID	OSM				LIF				CT-1				IL-6+R				IL-11 + sIL11R				IL-6				IL-11				CNTF				CLC				IL-27			
		FC	SD	p	FDR	FC	SD	p	FDR	FC	SD	p	FDR	FC	SD	p	FDR	FC	SD	p	FDR	FC	SD	p	FDR	FC	SD	p	FDR	FC	SD	p	FDR	FC	SD	p	FDR	FC	SD	p	FDR
**PDGF subunit A**	P04085	-0.11	0.22	0.550	0.653	0.20	0.10	0.108	0.215	0.15	0.14	0.274	0.488	0.36	0.23	0.153	0.233	0.19	0.15	0.210	0.395	0.28	0.28	0.288	0.512	0.30	0.30	0.294	0.482	0.02	0.34	0.949	0.981	-0.16	0.43	0.647	0.853	-0.08	0.44	0.827	0.996
**ALCAM**	Q13740	-0.14	0.10	0.190	0.324	0.14	0.14	0.309	0.424	0.08	0.14	0.479	0.589	0.29	0.22	0.204	0.269	0.11	0.18	0.453	0.647	0.21	0.28	0.41	0.583	0.13	0.22	0.486	0.595	-0.05	0.27	0.806	0.981	-0.25	0.26	0.298	0.853	-0.03	0.25	0.887	0.996
**U-PAR**	Q03405	-0.16	0.30	0.542	0.653	0.24	0.05	0.023	**0.095**	0.26	0.11	0.083	0.292	0.51	0.26	0.109	0.195	0.34	0.24	0.178	0.377	0.29	0.40	0.416	0.583	0.33	0.42	0.386	0.520	-0.05	0.38	0.876	0.981	-0.26	0.52	0.553	0.853	-0.09	0.48	0.821	0.996
**TNFRSF10C**	O14798	-0.21	0.07	0.050	0.168	-0.07	0.10	0.439	0.512	-0.19	0.24	0.387	0.535	0.45	0.18	0.072	0.160	0.06	0.11	0.526	0.695	0.39	0.28	0.183	0.512	0.02	0.04	0.632	0.714	-0.06	0.27	0.799	0.981	-0.23	0.39	0.495	0.853	-0.13	0.51	0.751	0.996
**TFF3**	Q07654	-0.23	0.13	0.124	0.249	-0.02	0.23	0.911	0.911	-0.14	0.14	0.297	0.488	0.46	0.15	0.048	0.136	0.00	0.16	0.997	0.997	0.33	0.30	0.260	0.512	0.05	0.34	0.858	0.884	-0.01	0.24	0.961	0.981	-0.09	0.41	0.780	0.853	-0.10	0.33	0.721	0.996
**MMP-2**	P08253	-0.23	0.09	0.065	0.171	0.17	0.05	0.046	0.123	0.13	0.14	0.322	0.501	0.41	0.21	0.108	0.195	0.16	0.14	0.249	0.405	0.33	0.35	0.321	0.513	0.29	0.29	0.297	0.482	-0.04	0.32	0.881	0.981	-0.29	0.56	0.544	0.853	-0.14	0.39	0.659	0.996
**IGFBP-2**	P18065	-0.24	0.31	0.390	0.504	0.28	0.13	0.097	0.200	0.2	0.32	0.473	0.589	0.72	0.42	0.136	0.222	0.32	0.24	0.203	0.395	0.50	0.42	0.236	0.512	0.44	0.35	0.216	0.459	0.06	0.52	0.888	0.981	-0.35	0.73	0.571	0.853	0.04	0.52	0.921	0.996
**PECAM-1**	P16284	-0.24	0.12	0.108	0.236	-0.11	0.10	0.239	0.356	-0.01	0.19	0.971	0.997	0.28	0.17	0.143	0.227	0.06	0.12	0.591	0.740	0.11	0.25	0.612	0.724	0.07	0.17	0.62	0.714	-0.14	0.18	0.394	0.927	-0.45	0.15	0.053	0.853	-0.17	0.18	0.313	0.996
**CD40**	P25942	-0.29	0.12	0.078	0.175	0.07	0.25	0.718	0.758	0.18	0.24	0.389	0.535	0.09	0.22	0.610	0.678	0.00	0.34	0.991	0.997	0.07	0.24	0.712	0.779	0.61	0.33	0.118	0.327	-0.02	0.12	0.809	0.981	-0.27	0.42	0.462	0.853	0.02	0.39	0.955	0.996
**CD163**	Q86VB7	-0.30	0.12	0.073	0.171	-0.22	0.19	0.247	0.361	-0.4	0.23	0.131	0.315	0.05	0.20	0.763	0.797	-0.34	0.17	0.099	0.325	-0.05	0.34	0.847	0.899	-0.62	0.15	0.028	0.252	0.10	0.08	0.221	0.618	0.07	0.22	0.704	0.853	0.07	0.18	0.614	0.996
**JAM-A**	Q9Y624	-0.31	0.33	0.309	0.441	0.05	0.27	0.830	0.842	-0.02	0.30	0.942	0.984	0.73	0.02	0.000	**0.014**	0.01	0.15	0.949	0.977	0.26	0.26	0.294	0.512	0.08	0.20	0.612	0.714	-0.27	0.38	0.425	0.954	-0.44	0.33	0.205	0.853	-0.12	0.31	0.635	0.996
**CST5**	P28325	-0.33	0.38	0.351	0.491	-0.16	0.33	0.572	0.626	-0.16	0.45	0.660	0.757	0.08	0.07	0.255	0.319	-0.35	0.20	0.130	0.325	0.12	0.23	0.535	0.668	0.16	0.55	0.711	0.754	0.21	0.07	0.048	0.241	-0.06	0.22	0.754	0.853	0.08	0.36	0.776	0.996
**PD-L1**	Q9NZQ7	-0.33	0.27	0.221	0.352	0.24	0.15	0.151	0.258	0.22	0.14	0.146	0.322	0.08	0.22	0.649	0.709	0.15	0.44	0.675	0.801	0.06	0.20	0.697	0.779	0.67	0.21	0.048	0.252	0.16	0.02	0.007	0.148	0.05	0.11	0.576	0.853	0.05	0.47	0.884	0.996
**LDL receptor**	P01130	-0.40	0.06	0.010	**0.087**	-0.03	0.05	0.486	0.540	-0.11	0.39	0.726	0.806	0.13	0.12	0.254	0.319	-0.23	0.19	0.217	0.395	0.23	0.19	0.235	0.512	-0.11	0.19	0.493	0.595	-0.04	0.47	0.926	0.981	0.02	0.52	0.969	0.969	0.00	0.44	0.996	0.996
**IL8**	P10145	-0.40	0.36	0.256	0.397	0.02	0.08	0.796	0.819	0.02	0.09	0.760	0.819	-0.29	0.21	0.187	0.256	-0.22	0.18	0.232	0.405	-0.01	0.11	0.946	0.946	0.45	0.33	0.187	0.409	0.05	0.11	0.570	0.981	-0.10	0.15	0.468	0.853	-0.05	0.45	0.900	0.996
**IL-6RA**	P08887	-0.41	0.23	0.133	0.258	-0.15	0.13	0.255	0.364	0.00	0.13	0.994	0.997	-	-	-	-	0.04	0.22	0.813	0.907	0.03	0.41	0.935	0.946	-0.03	0.39	0.924	0.938	-0.11	0.38	0.723	0.981	-0.37	0.88	0.610	0.853	-0.18	0.30	0.495	0.996
**PAI**	P05121	-0.48	0.19	0.071	0.171	0.15	0.08	0.110	0.215	0.10	0.11	0.304	0.488	0.19	0.14	0.201	0.269	0.10	0.12	0.387	0.603	0.20	0.20	0.300	0.512	0.28	0.30	0.315	0.489	0.03	0.13	0.782	0.981	-0.16	0.29	0.523	0.853	0.00	0.15	0.990	0.996
**MMP-10**	P09238	-0.60	0.44	0.189	0.324	0.19	0.25	0.399	0.484	0.11	0.16	0.429	0.577	-0.37	0.35	0.271	0.333	-0.32	0.47	0.436	0.636	0.12	0.27	0.586	0.708	0.71	0.21	0.042	0.252	0.21	0.05	0.027	0.215	-0.08	0.36	0.783	0.853	0.17	0.52	0.694	0.996
**4E-BP1**	Q13541	-0.69	0.10	0.011	**0.087**	-0.19	0.66	0.726	0.758	0.00	0.80	0.997	0.997	-0.44	0.38	0.236	0.306	0.16	0.92	0.826	0.907	-0.99	1.05	0.314	0.513	0.55	0.88	0.467	0.593	-0.53	0.64	0.361	0.903	-0.49	0.76	0.461	0.853	-0.28	0.79	0.663	0.996
**STAMBP**	O95630	-0.72	0.54	0.203	0.330	-0.46	0.71	0.454	0.521	-0.50	0.80	0.470	0.589	-0.20	0.26	0.399	0.466	-0.40	0.80	0.556	0.721	-0.30	0.43	0.434	0.595	-0.21	0.69	0.708	0.754	-0.08	0.27	0.701	0.981	0.04	0.23	0.824	0.871	-0.01	0.35	0.961	0.996
**t-PA**	P00750	-0.79	0.48	0.145	0.275	0.16	0.23	0.421	0.499	0.06	0.33	0.833	0.883	0.35	0.37	0.311	0.375	0.11	0.41	0.734	0.856	0.38	0.48	0.373	0.544	0.36	0.55	0.449	0.593	-0.02	0.49	0.969	0.981	-0.43	0.76	0.503	0.853	-0.05	0.62	0.920	0.996
**CSTB**	P04080	-0.82	0.24	0.041	0.145	-0.25	0.25	0.298	0.417	-0.29	0.30	0.307	0.488	0.35	0.15	0.080	0.163	-0.17	0.14	0.220	0.395	-0.03	0.38	0.922	0.946	-0.12	0.35	0.670	0.733	-0.36	0.10	0.039	0.241	-0.41	0.41	0.292	0.853	0.05	0.21	0.759	0.996
**CASP-3**	P42574	-0.86	0.15	0.014	**0.087**	-0.28	0.33	0.359	0.467	-0.27	0.35	0.390	0.535	0.08	0.22	0.667	0.718	-0.02	0.13	0.844	0.909	-0.35	0.74	0.568	0.698	0.09	0.50	0.831	0.868	-0.56	0.07	0.007	0.148	-0.59	0.39	0.166	0.853	-0.30	0.28	0.270	0.996
**uPA**	P00749	-0.91	0.35	0.068	0.171	-0.05	0.06	0.360	0.467	-0.08	0.07	0.253	0.466	-0.22	0.15	0.169	0.247	-0.26	0.23	0.242	0.405	0.03	0.30	0.914	0.946	0.41	0.35	0.239	0.479	-0.11	0.15	0.397	0.927	-0.35	0.31	0.257	0.853	0.00	0.39	0.990	0.996
**ADA**	P00813	-0.93	0.02	0.000	**0.016**	-0.14	0.46	0.701	0.755	-0.12	0.43	0.736	0.806	-0.37	0.15	0.073	0.160	-0.22	0.40	0.519	0.695	-0.44	0.40	0.264	0.512	0.43	0.51	0.359	0.509	-0.38	0.60	0.465	0.981	-0.55	0.57	0.307	0.853	-0.24	0.72	0.686	0.996
**AXL**	P30530	-0.96	0.38	0.070	0.171	0.14	0.16	0.335	0.450	0.06	0.24	0.737	0.806	0.02	0.36	0.942	0.942	0.01	0.25	0.947	0.977	0.25	0.45	0.504	0.641	0.31	0.49	0.465	0.593	0.09	0.39	0.766	0.981	-0.24	0.55	0.604	0.853	-0.03	0.48	0.937	0.996
**FABP4**	P15090	-1.12	0.40	0.059	0.171	-0.43	0.18	0.079	0.168	-0.67	0.40	0.143	0.322	-0.08	0.16	0.534	0.612	-0.25	0.34	0.415	0.631	0.06	0.19	0.702	0.779	0.00	0.09	0.992	0.992	-0.49	0.19	0.068	0.316	-0.61	0.53	0.242	0.853	-0.02	0.12	0.847	0.996
**CCL20**	P78556	-1.42	0.26	0.016	**0.087**	-0.80	0.60	0.201	0.312	-0.53	0.61	0.344	0.52	-0.37	0.03	0.004	**0.046**	-0.75	0.65	0.244	0.405	-0.20	0.17	0.247	0.512	0.31	0.19	0.144	0.327	0.07	0.32	0.793	0.981	0.11	0.32	0.671	0.853	0.25	0.27	0.331	0.996

Supplementary Table S2

	Enriched Function	p-value for enrichment	Regulated proteins that enriched the function	# Molecules
OSM	Activation of phagocytes	6,69E-11	MCP-1,CCL20,MCP-3,MCP-2,CSF-1,CXCL5,HGF,IL6,TRAIL	9
	Angiogenesis/Vasculogenesis	7,60E-08	ADA,CASP-3,MCP-1,MCP-3,CSF-1,CXCL5,HGF,IGFBP-1,IL6,TRAIL	10
	Binding	2,90E-08	CCL15,MCP-1,CXCL11,HGF,IGFBP-1,IL6	6
	Branching of cells	7,55E-05	CASP-3,MCP-1,4E-BP1,HGF,IL6	5
	Cell movement	2,98E-13	ADA,CCL15,MCP-1,CCL20,CCL23,MCP-3,MCP-2,CSF-1,CXCL11,CXCL5,HGF,IL6,TRAIL	13
	Cell proliferation	3,20E-06	MCP-1,CSF-1,4E-BP1,HGF,IGFBP-1,IL6,TRAIL	7
	Chemotaxis	3,26E-12	CCL15,MCP-1,CCL20,CCL23,MCP-3,MCP-2,CSF-1,CXCL11,CXCL5,HGF,IL6	11
	Differentiation	2,40E-06	MCP-1,CSF-1,HGF,IL6,TRAIL	5
	Endocytosis	4,83E-05	MCP-3,CSF-1,HGF,IL6,TRAIL	5
	Growth	8,85E-06	MCP-1,CSF-1,CXCL5,4E-BP1,HGF,IL6	6
	Invasion	1,62E-06	CASP-3,MCP-1,CSF-1,CXCL11,CXCL5,4E-BP1,HGF,IL6,TRAIL	9
	Migration	8,95E-12	TR-AP,ADA,CCL15,MCP-1,CCL20,CCL23,MCP-3,MCP-2,CSF-1,CXCL11,CXCL5,4E-BP1,HGF,IGFBP-1,IL6,TRAIL	16
	Recruitment	9,98E-13	CCL15,MCP-1,CCL20,CCL23,MCP-3,CSF-1,CXCL11,CXCL5,HGF,IL6	10
	Stimulation	6,51E-14	MCP-1,CCL20,MCP-3,CSF-1,CXCL11,CXCL5,HGF,IL6,TRAIL	9
LIF	Adhesion	1,18E-10	MCP-3,CSF-1,CXCL11,HGF,IL6,LAP TGF-beta-1,OPG,TWEAK	8
	Angiogenesis/Vasculogenesis	6,06E-08	MCP-3,CSF-1,CXCL5,HGF,IGFBP7,IL6,LAP TGF-beta-1,TRAIL,TWEAK	9
	Attraction	7,89E-09	MCP-3,CSF-1,CXCL11,CXCL5,LAP TGF-beta-1	5
	Binding	3,79E-12	MCP-3,CSF-1,CXCL11,HGF,IL-18R1,IL6,LAP TGF-beta-1,OPG,TWEAK	9
	Cell movement	4,65E-12	CCL23,MCP-3,CSF-1,CXCL11,CXCL5,HGF,IL6,LAP TGF-beta-1,OPG,TRAIL,TWEAK	11
	Cell proliferation	1,20E-10	CSF-1,HGF,IGFBP7,IL6,LAP TGF-beta-1,OPG,TRAIL,TWEAK	8
	Chemotaxis	6,98E-09	CCL23,MCP-3,CSF-1,CXCL11,CXCL5,HGF,IL6,LAP TGF-beta-1	8
	Development of vasculature	6,77E-09	MCP-3,CSF-1,CXCL5,HGF,IGFBP7,IL6,LAP TGF-beta-1,OPG,TRAIL,TWEAK	10
	Differentiation	5,84E-07	CSF-1,HGF,IL6,LAP TGF-beta-1,TRAIL	5
	Endocytosis	4,36E-07	MCP-3,CSF-1,HGF,IL6,LAP TGF-beta-1,TRAIL	6
	Mobilization of Ca2+	2,85E-06	CCL23,MCP-3,CXCL5,IL6,LAP TGF-beta-1	5
	Formation	1,32E-07	MCP-3,HGF,IL6,LAP TGF-beta-1,TRAIL	5
	Induction	2,06E-10	CSF-1,HGF,IL-18R1,IL6,LAP TGF-beta-1,TRAIL	6
	Invasion	1,00E-08	CSF-1,CXCL5,HGF,IL6,LAP TGF-beta-1	5
	Migration	2,75E-10	TR-AP,CCL23,MCP-3,CSF-1,CXCL11,CXCL5,HGF,IGFBP7,IL6,LAP TGF-beta-1,OPG,TRAIL,TWEAK	13
	Recruitment	2,22E-12	CCL23,MCP-3,CSF-1,CXCL11,CXCL5,HGF,IL6,LAP TGF-beta-1,TWEAK	9
	Stimulation	2,78E-15	MCP-3,CSF-1,CXCL11,CXCL5,HGF,IL-18R1,IL6,LAP TGF-beta-1,TRAIL	9
CT-1	Adhesion	5,15E-11	MCP-3,CSF-1,CXCL11,HGF,IL6,LAP TGF-beta-1,OPG,TWEAK	8
	Angiogenesis/Vasculogenesis	2,42E-06	MCP-3,CSF-1,HGF,IL6,LAP TGF-beta-1,TRAIL,TWEAK	7
	Binding	1,38E-12	MCP-3,CSF-1,CXCL11,HGF,IL-18R1,IL6,LAP TGF-beta-1,OPG,TWEAK	9
	Cell movement	1,05E-12	CCL23,MCP-3,CDCP1,CSF-1,CXCL11,HGF,IL6,LAP TGF-beta-1,OPG,TRAIL,TWEAK	11
	Cell proliferation	3,22E-09	CSF-1,HGF,IL6,LAP TGF-beta-1,OPG,TRAIL,TWEAK	7
	Chemotaxis	3,09E-09	CCL23,MCP-3,CDCP1,CSF-1,CXCL11,HGF,IL6,LAP TGF-beta-1	8
	Development of vasculature	1,32E-06	MCP-3,CSF-1,HGF,IL6,LAP TGF-beta-1,OPG,TRAIL,TWEAK	8
	Differentiation	3,79E-07	CSF-1,HGF,IL6,LAP TGF-beta-1,TRAIL	5
	Endocytosis	2,54E-07	MCP-3,CSF-1,HGF,IL6,LAP TGF-beta-1,TRAIL	6
	Formation	8,53E-08	MCP-3,HGF,IL6,LAP TGF-beta-1,TRAIL	5
	Induction	1,18E-10	CSF-1,HGF,IL-18R1,IL6,LAP TGF-beta-1,TRAIL	6
	Invasion	1,32E-05	CDCP1,CSF-1,CXCL11,HGF,IL6,LAP TGF-beta-1,TRAIL	7
	Migration	1,68E-09	TR-AP,CCL23,MCP-3,CDCP1,CSF-1,CXCL11,HGF,IL6,LAP TGF-beta-1,OPG,TRAIL,TWEAK	12
	Recruitment	6,54E-11	CCL23,MCP-3,CSF-1,CXCL11,HGF,IL6,LAP TGF-beta-1,TWEAK	8
	Stimulation	1,74E-13	MCP-3,CSF-1,CXCL11,HGF,IL-18R1,IL6,LAP TGF-beta-1,TRAIL	8

Supplementary Table S2 (continued)

	Enriched Function	p-value for enrichment	Regulated proteins that enriched the function	# Molecules
**IL-6 +sIL-6R**	Angiogenesis/Vasculogenesis	4,12E-09	MCP-1,MCP-3,CSF-1,CXCL5,JAM-A,GRN,HGF,IGFBP-1,IGFBP7,TNF-R1,TRAIL	11
	Attraction	3,71E-10	MCP-1,MCP-3,CSF-1,CXCL11,CXCL16,CXCL5	6
	Cell death	6,40E-06	CSF-1,CXCL11,GDF15,HGF,OPG,TRAIL	6
	Cell movement	9,52E-17	MCP-1,CCL23,MCP-3,CSF-1,CXCL11,CXCL16,CXCL5,JAM-A,GDF15,GRN,HGF,IL-15RA,OPG,TNF-R1,TRAIL	15
	Cell proliferation	1,62E-09	MCP-1,CSF-1,GRN,HGF,IGFBP7,OPG,TNF-R1,TRAIL	8
	Chemotaxis	1,22E-10	MCP-1,CCL23,MCP-3,CSF-1,CXCL11,CXCL16,CXCL5,GRN,HGF,TNF-R1	10
	Development of vasculature	6,62E-10	MCP-1,MCP-3,CSF-1,CXCL5,JAM-A,GRN,HGF,IGFBP-1,IGFBP7,OPG,TNF-R1,TRAIL	12
	Differentiation	2,40E-06	MCP-1,CSF-1,HGF,TNF-R1,TRAIL	5
	Endocytosis	4,61E-05	MCP-3,CSF-1,GRN,HGF,TNF-R1,TRAIL	6
	Invasion	1,62E-06	MCP-1,CSF-1,CXCL11,CXCL5,JAM-A,GDF15,GRN,HGF,TRAIL	9
	Migration	1,83E-15	TR-AP,MCP-1,CCL23,MCP-3,CSF-1,CXCL11,CXCL16,CXCL5,JAM-A,GDF15,GRN,HGF,IGFBP-1,IGFBP7,IL-15RA,OPG,TNF-R1,TRAIL	18
	Mobilization of Ca2+	7,24E-07	MCP-1,CCL23,MCP-3,CXCL11,CXCL16,CXCL5	6
	Organization	2,23E-05	MCP-1,MCP-3,CSF-1,JAM-A,GDF15,GRN,HGF,TNF-R1,TRAIL	9
	Proliferation	3,64E-08	MCP-1,CSF-1,JAM-A,GRN,HGF,IGFBP-1,TRAIL	7
	Recruitment	1,74E-07	MCP-1,CCL23,MCP-3,CSF-1,CXCL5,TNF-R1	6
	Stimulation	3,97E-10	MCP-1,MCP-3,CSF-1,CXCL11,CXCL5,HGF,TRAIL	7
	Synthesis of lipid	2,71E-05	MCP-1,CSF-1,GDF15,HGF,IGFBP7,TNF-R1,TRAIL	7
**IL-11 +sIL-11R**	Angiogenesis/Vasculogenesis	8,56E-07	MCP-1,MCP-3,HGF,IGFBP7,IL6,TRAIL	6
	Cell movement	5,54E-09	MCP-1,CCL23,MCP-3,HGF,IL6	5
	Stimulation	7,00E-09	MCP-1,MCP-3,HGF,IL6,TRAIL	5
	Cell proliferation	2,44E-07	MCP-1,HGF,IGFBP7,IL6,TRAIL	5
	Migration	2,84E-07	TR-AP,MCP-1,CCL23,MCP-3,HGF,IGFBP7,IL6,TRAIL	8
	Chemotaxis	3,12E-06	MCP-1,CCL23,MCP-3,HGF,IL6	5
	Synthesis of lipid	2,38E-05	MCP-1,HGF,IGFBP7,IL6,TRAIL	5
	Homeostasis	5,15E-05	MCP-1,CCL23,MCP-3,HGF,IL6,TRAIL	6
**IL-6**	Chemotaxis	6,10E-08	CCL23,MCP-3,CXCL11,CXCL5,HGF	5
	Cell movement	2,26E-06	CCL23,MCP-3,CXCL11,CXCL5,HGF	5
**IL-11**	Adhesion	6,34E-10	MCP-3,CSF-1,CXCL11,HGF,LAP TGF-beta-1,OPG,TRANCE	7
	Cell movement	3,14E-06	CCL23,MCP-3,CSF-1,CXCL11,HGF,LAP TGF-beta-1,OPG	7
	Cell proliferation	1,91E-06	CSF-1,HGF,LAP TGF-beta-1,OPG,TRANCE	5
	Chemotaxis	2,29E-08	CCL23,MCP-3,CSF-1,CXCL11,HGF,LAP TGF-beta-1,TRANCE	7
	Formation	2,39E-06	MCP-3,HGF,LAP TGF-beta-1,TRANCE	4
	Invasion	2,90E-06	PD-L1,CSF-1,CXCL11,HGF,MMP-10,LAP TGF-beta-1,TRANCE	7
	Metastasis	2,34E-06	PD-L1,CSF-1,HGF,MMP-10,LAP TGF-beta-1,OPG,TRANCE	7
	Migration	2,14E-05	MCP-3,CSF-1,CXCL11,HGF,LAP TGF-beta-1,OPG	6
